# A CoA‐Transferase and Acyl‐CoA Dehydrogenase Convert 2‐(Carboxymethyl)cyclohexane‐1‐Carboxyl‐CoA During Anaerobic Naphthalene Degradation

**DOI:** 10.1111/1462-2920.70013

**Published:** 2024-12-19

**Authors:** Yachao Kong, Jan Riebe, Malte Feßner, Torsten Schaller, Christoph Wölper, Florian Stappert, Sven W. Meckelmann, Matthias Krajnc, Philip Weyrauch, Oliver J. Schmitz, Christian Merten, Jochen Niemeyer, Xiaoke Hu, Rainer U. Meckenstock

**Affiliations:** ^1^ Key Laboratory of Coastal Biology and Bioresource Utilization Yantai Institute of Coastal Zone Research, Chinese Academy of Sciences Yantai China; ^2^ Institute for Environmental Microbiology and Biotechnology, Aquatic Microbiology University of Duisburg‐Essen Essen Germany; ^3^ College of Resource and Environment University of Chinese Academy of Sciences Beijing China; ^4^ Organic Chemistry, Faculty of Chemistry and Center for Nanointegration, Duisburg‐Essen (CENIDE) University of Duisburg‐Essen Essen Germany; ^5^ Organic Chemistry II, Faculty of Chemistry and Biochemistry Ruhr Universität Bochum Bochum Germany; ^6^ Organic Chemistry, Faculty of Chemistry University of Duisburg‐Essen Essen Germany; ^7^ Inorganic Chemistry, Faculty of Chemistry University of Duisburg‐Essen Essen Germany; ^8^ Applied Analytical Chemistry University of Duisburg‐Essen Essen Germany; ^9^ Qingdao Marine Science and Technology Center Laboratory for Marine Biology and Biotechnology Qingdao China

## Abstract

The CoA thioester of 2‐(carboxymethyl)cyclohexane‐1‐carboxylic acid has been identified as a metabolite in anaerobic naphthalene degradation by the sulfate‐reducing culture N47. This study identified and characterised two acyl‐CoA dehydrogenases (ThnO/ThnT) and an intramolecular CoA‐transferase (ThnP) encoded within the substrate‐induced thn operon, which contains genes for anaerobic degradation of naphthalene. ThnP is a CoA transferase belonging to the family I (Cat 1 subgroup) that catalyses the intramolecular CoA transfer from the carboxyl group of 2‐(carboxymethyl)cyclohexane‐1‐carboxyl‐CoA to its carboxymethyl moiety, forming 2‐carboxycyclohexylacetyl‐CoA. Neither acetyl‐CoA nor succinyl‐CoA functions as an exogenous CoA donor for this reaction. The flavin‐dependent homotetrameric dehydrogenase ThnO is specific for (1R,2R)‐2‐carboxycyclohexylacetyl‐CoA with an apparent *K*_m_ value of 61.5 μM, whereas ThnT is a promiscuous enzyme catalysing the same reaction at lower rates. Identifying these three enzymes confirmed the involvement of the thn gene cluster in the anaerobic naphthalene degradation pathway. This study establishes a modified metabolic pathway for anaerobic naphthalene degradation upstream of 2‐(carboxymethyl)cyclohexane‐1‐carboxyl‐CoA and provides further insight into the subsequent second‐ring cleavage reaction.

## Graphical abstract

We identified a CoA‐transferase and an acyl‐CoA dehydrogenase involved in converting 2‐(carboxymethyl)cyclohexane‐1‐carboxyl‐CoA during anaerobic naphthalene degradation. This confirms the *thn* gene cluster's role in this process. We also modified the metabolic pathway upstream and provided further insight into the second‐ring cleavage reaction.

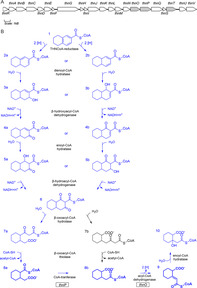

## 1 Introduction

Naphthalene is a hazardous environmental pollutant originating from natural and anthropogenic sources such as oil spills (Lawal 2017). Due to the chemical stability and poor bioavailability naphthalene appears recalcitrant in the environment and accumulates especially in anoxic sediments (Haritash and Kaushik 2009). Naphthalene can be well degraded by aerobic microorganisms (Gibson and Parales 2000; Habe and Omori 2003) but knowledge about anaerobic degradation of naphthalene is scarce (Meckenstock et al. 2016).

**FIGURE 1:**
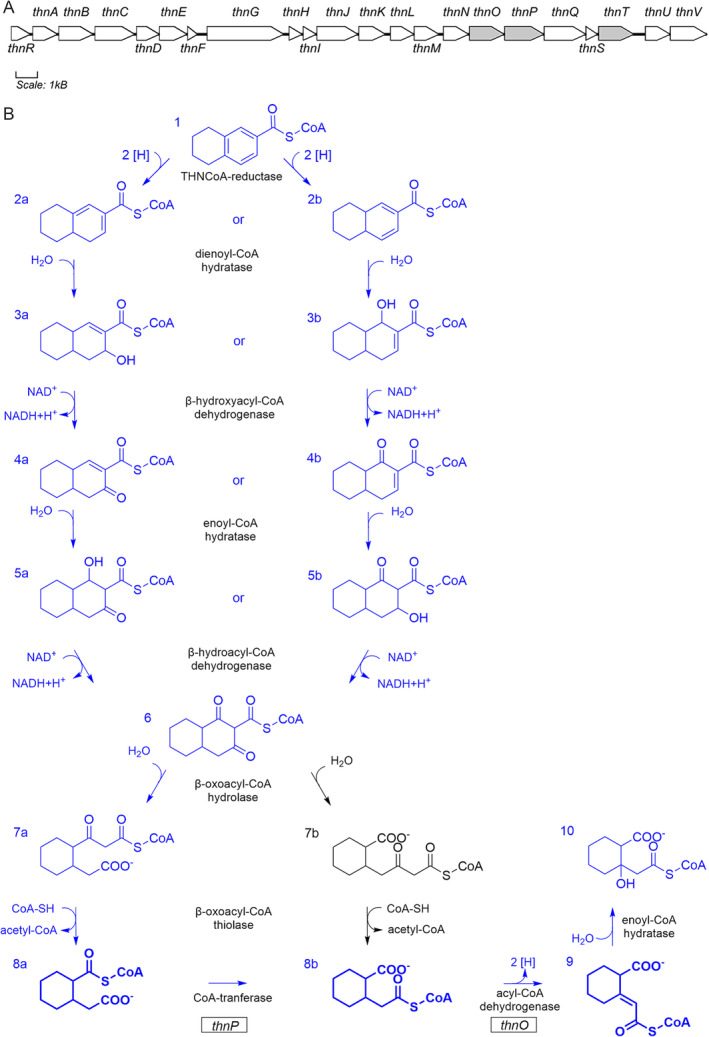
(A) Organisation of the *thn* gene cluster in the sulfate‐reducing enrichment culture N47. Genes labelled in grey are subject to the present paper. (B) Schematic view of the upstream part of the anaerobic naphthalene degradation pathway producing 2‐(carboxymethyl)cyclohexane‐1‐carboxyl‐CoA (8a) and its conversion by CoA‐transferase ThnP and acyl‐CoA dehydrogenase ThnO. The two possible isomers of the product of tetrahydro‐naphthoyl‐CoA reductase, 4,4a,5,6,7,8‐hexahydro‐2‐naphthoyl‐CoA (2a) and 4a,5,6,7,8,8a‐hexahydro‐2‐naphthoyl‐CoA (2b), would lead to different downstream pathways yielding either 2‐(carboxymethyl)cyclohexane‐1‐carboxyl‐CoA (8a) or 2‐(2‐carboxycyclohexyl)‐acetyl‐CoA (8b). The pathway in blue colour is proposed to be correct according to the experimental results of the conversion of 2‐(carboxymethyl)cyclohexane‐1‐carboxyl‐CoA by ThnP and ThnO in this study (adapted from reference Weyrauch et al. 2020). Reactions catalysed by ThnP and ThnO are highlighted in bold print.

The sulfate‐reducing, highly enriched culture N47 is capable of anaerobic growth with naphthalene as the sole electron and carbon source (Meckenstock et al. 2000). Anaerobic naphthalene degradation is initialized by carboxylation of naphthalene to 2‐naphthoic acid (Zhang and Young 1997; Mouttaki, Johannes, and Meckenstock 2012; Heker, Haberhauer, and Meckenstock 2023) followed by a CoA‐ligase reaction forming 2‐naphthoyl‐CoA (Arnold et al. 2021). Next, two type III aryl‐CoA reductases, namely 2‐naphthoyl‐CoA reductase and dihydro‐2‐naphthoyl‐CoA reductase, catalyse the stepwise reduction of 2‐naphthoyl‐CoA to 5,6,7,8‐tetrahydro‐2‐naphthoyl‐CoA (THN‐CoA) (Eberlein, Estelmann et al. 2013a; Eberlein, Johannes et al. 2013b). Then, a dearomatising type I aryl‐CoA reductase reduces the remaining aromatic ring to hexahydro‐2‐naphthoyl‐CoA (Estelmann et al. 2015). The following metabolic steps are β‐oxidation‐like reactions leading to cleavage of ring I of naphthoic acid producing a CoA thioester of *cis*‐2‐(carboxymethyl)cyclohexane‐1‐carboxylic acid (Figure 1B) (Annweiler, Michaelis, and Meckenstock 2002; Weyrauch et al. 2020). The fate of the intermediate 2‐(carboxymethyl)cyclohexane‐1‐carboxylic acid coenzyme A thioester was investigated earlier in cell free extracts of the naphthalene‐degrading strains N47 and NaphS2. The reactions proceed via an unsaturation of the carboxymethyl side‐chain and water addition to the β‐position yielding a tertiary hydroxyl group producing 2‐(1‐hydroxy‐2‐carboxycyclohexyl)acetyl‐CoA (Weyrauch et al. 2017).

Expression of a gene‐cluster coding for candidate enzymes of the THN‐CoA reduction and of the downstream pathway was found in 2‐methylnaphthalene‐grown cells of N47 by proteogenomic analyses (Selesi et al. 2010; Bergmann, Selesi, and Meckenstock 2011). A similar gene cluster was found in the marine naphthalene‐degrading strain NaphS2 grown with 2‐naphthoate versus benzoate or pyruvate (DiDonato et al. 2010). Both gene clusters code for putative β‐oxidation‐related enzymes such as hydrolases, dehydrogenases and CoA‐transferases but the function of the individual gene products remained unclear.

Here, we aimed to verify the role of the *thn* gene cluster in the anaerobic naphthalene degradation pathway by identifying enzymes and the respective reactions involved in the degradation of 2‐(carboxymethyl)cyclohexane‐1‐carboxylic acid coenzyme A thioester. To this end, we cloned and expressed candidate genes for three enzyme reactions in *Escherichia coli*, purified the enzymes and elucidated the respective enzyme reactions.

## 2 Experimental Procedures

### 2.1 Growth of Bacterial Cells

The enrichment culture N47 (Meckenstock et al. 2000) was cultivated anaerobically with 150 mL artificial freshwater medium in 200‐mL serum bottles closed with blue butyl stoppers (Glasgerätebau Ochs, Bovenden/Lenglern, Germany) as described before (Widdel and Pfennig 1981; Weyrauch et al. 2017). 3 mL of a 1.5% (w/v) naphthalene solution in 2,2,4,4,6,8,8‐heptamethylnonane was added as the sole source of carbon and electrons and 20 mM sulfate was added as electron acceptor. After being incubated at 30°C in the dark for 6 weeks, 150 mL of pre‐culture was inoculated into 1.6 L of the same medium in 2‐l Schott bottles sealed with black butyl stoppers (Glasgerätebau Ochs) and incubated for 6 weeks before cells were harvested.

### 2.2 Plasmid Construction

The genes *thnO* (N47_E41350), *thnT* (N47_E41310) and *thnP* (N47_E41340) were amplified from the N47 genome (Gene bank: FR695877.1) which were referred to as ORF42, ORF46 and ORF43 in a previous work (Table S1) (Selesi et al. 2010). *thnO* and *thnT* genes were expressed with either His6‐tag or Twin‐Strep‐tag at the C‐terminus. For gene *thnP*, only a plasmid with a His6‐tag at the C‐terminus was constructed. The genomic DNA of strain N47 was isolated from a mid‐exponential culture as described previously (Lueders, Manefield, and Friedrich 2004). Target genes were amplified from genomic DNA of N47 with Phusion Hot Start II High‐Fidelity DNA Polymerase (Thermo Fisher Scientific, Ulm, Germany) following the manufacturer's protocol and using the primer pairs listed in Table S2. The amplicons were ligated as amplification template into the pETM‐13 vector (European Molecular Biology Laboratory, EMBL, Heidelberg, Germany) via the NcoI and XhoI restriction sites, resulting in genes with a His6‐tag at the C‐terminus. Then, *thnO* and *thnT* were cloned into the pASG‐IBA103 vector (IBA Lifesciences, Göttingen, Germany) following the manufacturer's protocol, resulting in an expression vector for Twin‐Strep‐tag proteins.

### 2.3 Heterologous Production in *Escherichia coli* and Purification

Gene expression for His‐tagged proteins was carried out in *Escherichia coli* Rosetta 2(DE3) (Merck Millipore, Darmstadt, Germany). Pre‐cultures were grown in Lysogeny broth medium (LB) containing 100 μg mL^−1^ kanamycin and 34 μg mL^−1^ chloramphenicol incubated at 37°C and 200 rpm. Auto‐induction medium ZYM‐5052 (Studier 2005) was inoculated with 1% (v/v) of pre‐culture. The cultures were grown at 20°C and 120 rpm and gene production was induced when the cell density reached an OD_600nm_ of 0.4 to 0.6. Cells were harvested in a 500‐mL centrifuge beaker (Corning, Oneonta, USA) by centrifugation for 30 min at 3200 × g and 4°C (Centrifuge 5810 R, Eppendorf, Hamburg, Germany). The cell pellets were resuspended in 8 mL of buffer containing 50 mM sodium phosphate at pH 8.0, 300 mM NaCl and 5 μM imidazole and opened by French press (Thermo Fisher, Dreieich, Germany) and a French press cell with 35 mL volume (Thermo Fisher) at approximately 8 × 10^6^ Pa. Cell debris was pelleted in 5‐mL tubes (Sarstedt, Nümbrecht, Germany) by centrifugation for 30 min at 18,200 × g and 4°C (Centrifuge 5430R, Eppendorf) and the supernatant was applied to a His GraviTrap TALON column (Cytiva, Amersham, UK) according to the manufacturer's instructions. After washing with 10 mL of 20 mM imidazole, 3 mL of elution buffer containing 100 mM imidazole was used to elute target proteins. Imidazole was removed from eluted proteins with a PD‐10 desalting column (Cytiva) according to the manufacturer's instructions. All steps were performed in ambient air. For purification of protein ThnP, 0.05% (v/v) 2‐mercaptoethanol was added to all buffers.

Gene expression of proteins with Twin‐Strep‐tag was carried out in *E. coli* BL21(DE3) (New England Biolabs, Frankfurt am Main, Germany). Cells were grown overnight at 22°C and 130 rpm in LB medium containing 0.2 μg ml^−1^ anhydrotetracycline as inducer and 100 μg ml^−1^ ampicillin. Buffer for suspending cells and column washing steps contained 100 mM Tris/HCl, pH 8.0, 150 mM NaCl and 1 mM EDTA. Elution buffer contained an additional 50 mM biotin. Strep‐Tactin columns (IBA Lifesciences) were used for protein purification according to the manufacturer's instructions.

### 2.4 Protein Size Determination

The molecular masses of the purified polypeptides were determined by reducing sodium dodecyl sulfate−polyacrylamide gel electrophoresis (SDS − PAGE) (Bio‐Rad, Feldkirchen, Germany) and Blue Native PAGE was performed to estimate the native molecular weight (Invitrogen, CA, USA) following the manufacturer's protocol. The molecular mass of the native protein complex was furthermore estimated by size exclusion chromatography with an Ekta purifier (GE Healthcare, Uppsala, Sweden), equipped with a Superose 6 column (10 × 300 mm, Cytiva) that was equilibrated with 50 mM Tris/HCl buffer, pH 8.0 and 250 mM NaCl. The column was eluted with the same buffer at a flow rate of 0.5 mL min^−1^. The molecular size of the polymeric complex was determined by comparison with reference standards (catalogue number 28403841, 28403842; Cytiva).

### 2.5 Identification and Quantification of a Flavin Cofactor of ThnO

A 15.8 μM (0.66 mg mL^−1^) solution of purified ThnO in 1 mL of 50 mM potassium phosphate buffer was prepared in a quartz cuvette (catalogue number Y033.1; Carl Roth, Karlsruhe, Germany) sealed with a stopper inside a glove box (LabStar ECO, Garching, Germany) with an atmosphere of 100% N_2_. UV–vis spectra of the oxidised protein were taken with a Tecan UVs spectrophotometer (infinite M200 Pro, Tecan, Grödig Austria) between 230 and 700 nm. 80 μM sodium dithionite was added to the protein solution with a syringe through the stopper and measured immediately with UV–vis spectroscopy to analyse the reduced FAD cofactor.

The FAD content in the protein was determined with LC–MS (LC 2040C, LCMS 2020; Shimadzu, Duisburg, Germany) (see below) by comparison to an external FAD standard curve. A 158 μM (6.6 mg mL^−1^) solution of ThnO in 150 μL elution buffer (same as the elution buffer for ThnO purification) was denatured by adding 750 μL methanol which precipitated all polypeptides. After removing the precipitated protein by centrifugation in a 1.5‐mL Eppendorf reaction tube at 18,200 × g, the supernatant was analysed by LC–MS.

### 2.6 Anaerobic Preparation of the Cell Free Extract of Strain N47

The preparation of cell free extract was performed in a glove box with an N_2_ atmosphere. A 1.6 L culture was transferred into gas‐tight centrifuge bottles (4 × 400 mL) in the glove box and cells were harvested by centrifugation for 30 min at 3200 × g and 4°C. The supernatant was discarded and the cell pellets were suspended in 3 mL of 100 mM MOPS (4‐morpholinepropanesulfonic acid)/KOH buffer pH 7.3 containing 15 mM MgCl_2_. Then, the cells were transferred into 1.5‐mL Eppendorf reaction tubes, centrifuged again and suspended in the same buffer. The cells were opened anoxically with a French press (Thermo Fisher) and a French press cell with 3.5 mL volume (G. Heinemann, Schwäbisch Gmünd, Germany) operated at 7 × 10^6^ Pa. The cell extract was collected in a stoppered glass vial that was flushed with N_2_/CO_2_ gas (80/20) and transferred to a 2‐mL airtight reaction tube (Sarstedt) in the glove box. Cell debris was separated by centrifugation for 90 min at 18,200 × g and 4°C.

### 2.7 Synthesis of Potential Metabolites and Elucidation of Enantiomeric Structure

Racemic *cis*‐isomer (1*R*,2*R*/1*S*,2*S*) and *trans*‐isomer (1*R*,2*S*/1*S*,2*R*) of 2‐(carboxymethyl)cyclohexane‐1‐carboxylic acid were synthesised earlier as described before (Weyrauch et al. 2017). 5,6,7,8‐tetrahydro‐2‐naphthoyl‐CoA and the CoA thioesters of racemic *cis*‐ and *trans*‐isomer of 2‐(carboxymethyl)cyclohexane‐1‐carboxylic acid were synthesised from the free acids via carbonyldiimidazole activation (Kawaguchi, Yoshimura, and Okuda 1981) and were purified as described earlier (Weyrauch et al. 2017). The 2‐(carboxymethyl)cyclohexane‐1‐carboxylic acid CoA thioesters intermediate (*m*/*z* = 936) was also synthesised anaerobically from THN‐CoA with N47 cell free extract via a THN‐CoA reductase assay with NADPH as electron donor (Weyrauch et al. 2020). Resolution of enantiomers of *cis*‐2‐(carboxymethyl)cyclohexane‐1‐carboxylic acid was performed by a derivatization with enantiopure alcohol (*R*)‐2‐phenyl‐1‐propanol (Neises and Steglich 1978) to form a mixture of diastereomeric diesters for separation (Fogassy et al. 2006; Heike Lorenz and Seidel‐Morgenstern 2014). A brief description of the approach is provided here; full experimental details can be found in Appendix S1.

### 2.8 Discontinuous Assays for Determination of Enzyme Activities

Enzyme activities were determined under strictly anoxic conditions in 1.5‐mL reaction tubes at 30°C and 900 rpm in a ThermoMixer C inside a glove box with an N_2_ atmosphere. 50 mM potassium phosphate buffer, pH 8.0 was used as the standard buffer for the heterologously produced enzymes. Alternatively, a buffer of 100 mM MOPS, 15 mM MgCl_2_, pH 7.3 (KOH) was used for assays with N47 cell free extract. The acyl‐CoA dehydrogenase assays were performed with a 200 μL reaction mixture containing either approximately 0.4 mM of the natural 2‐(carboxymethyl)cyclohexane‐1‐carboxylic acid CoA thioesters produced with cell‐free extract or 0.2 mM of chemically synthesised *cis*‐/*trans*‐2‐(carboxymethyl)cyclohexane‐1‐carboxylic acid CoA thioesters. 2.5 mM ferrocenium hexafluorophosphate and 2.5 mM phenazine methosulfate were used as artificial electron acceptors and redox mediators (Engel and Massey 1971; Lehman and Thorpe 1990). The acyl‐CoA dehydrogenase reactions were started by the addition of 22.5 μL expressed ThnO (4–23 μg) or ThnT (4–53 μg), or 60 μL cell free extract (resulting in a final protein concentration of 1.3–2.0 mg/mL). Alternatively, 32 μM of the natural 2‐(carboxymethyl)cyclohexane‐1‐carboxylic acid CoA thioesters or 0.2 mM of chemically synthesised *cis*‐/*trans*‐2‐(carboxymethyl)cyclohexane‐1‐carboxylic acid CoA thioesters were used as substrates, a mixture of ThnO (9 to 112 μg) and ThnP (9 to 24 μg) were used for the combined CoA‐transferase and acyl‐CoA dehydrogenase assay. The respective protein amount is provided in the descriptions of the experiments.

*cis*‐2‐(carboxymethyl)cyclohexane‐1‐carboxylic acid, 2‐(carboxymethyl)cyclohex‐1‐enecarboxylic acid and *cis*‐(E)‐3‐(2‐(carboxymethyl)cyclohexyl) acrylic acid were used to test the intermolecular CoA transfer activity of ThnP using either acetyl‐CoA or succinyl‐CoA as CoA donor.

30 μL samples were taken at different time points and a double volume of methanol was added to stop the reaction. Afterward, the samples were centrifuged for 45 min at 18,200 × g and 4°C before the supernatant was analysed by LC–MS.

### 2.9 LC–MS Analysis

Analysis of enzyme reactions was performed by with a Shimadzu high‐performance liquid chromatography LC‐2040C system coupled to a LCMS‐2020 single quadrupole mass‐spectrometer (Shimadzu) (LC–MS). Samples were separated via a Nucleodur C18 Gravity‐SB column (100 × 3 mm, 5 μm particle size, Macherey‐Nagel) at 35°C. Compounds were eluted with an increasing gradient of acetonitrile (buffer B) in a 0.1% (w/v) ammonium formate buffer (pH 6.5) (buffer A) at a flow rate of 0.4 mL min^−1^ from 5% to 35% over 15 min. The mass spectrometer was operated with an ESI system in positive mode. The voltage of the ESI system was set to 4.5 kV, the flow of the nebulizing gas was 1.5 L min^−1^, and drying gas flow was 12 L min^−1^.

### 2.10 Steady‐State Kinetics of ThnO

Steady‐state kinetic analysis of ThnO was conducted by enzyme tests with *cis*‐2‐(carboxymethyl)cyclohexane‐1‐carboxylic acid CoA thioesters concentrations ranging from 50 to 1500 μM, using 12.8 μg enzyme purified from *E. coli* with Strep‐tag. Considering the proportions of 2‐carboxycyclohexylacetyl‐CoA and 2‐(carboxymethyl)cyclohexane‐1‐carboxyl‐CoA, and (1*S*,2*S*)‐ and (1*R*,2*R*)‐isomers in the substrate, the real substrate concentrations ranged from 10 to 296 μM. Assays were performed with a 1 mL reaction mixture containing 0.2 mM ferrocenium hexafluorophosphate in quartz cuvettes at 30°C and anoxic condition as mentioned before. The initial rates of product formation were determined following the reduction of ferrocenium hexafluorophosphate at 300 nm with a Cary 50 UV–Visible Spectrophotometer (Varian, Darmstadt, Germany) and *V*_max_ and *K*_m_ values were calculated with the Michaelis–Menten equation.

### 2.11 Absolute Configuration Determination of *cis* ‐2‐(Carboxymethyl)cyclohexane‐1‐Carboxylic Acid by VCD Spectroscopy and X‐Ray Crystallography

Infrared spectroscopy (IR) and vibrational circular dichroism (VCD) spectra of the enantiopure non‐CoA derivative of the real substrate of dehydrogenase ThnO were recorded with a Bruker Invenio‐R/PMA 50 VCD spectrometer. The IR and VCD spectra were simulated from the single‐conformer spectra for final comparison with the experimental data. Additionally, crystals suitable for single crystal X‐ray analysis were obtained after slow evaporation of a solution of the enantiopure non‐CoA derivative of the real substrate, and data were collected on a Bruker D8 Venture at 100(2) K. The structure was subsequently solved. Detailed procedures for VCD spectroscopy and X‐ray crystallography are provided in Data S1.

### 2.12 Derivatization and GC–MS to Determine the Position of the CoA Thioester

To identify if the CoA thioester is attached at the carboxyl or the carboxymethyl residue in the isomeric mixture of 2‐(carboxymethyl)cyclohexane‐1‐carboxylic acid CoA thioesters, produced by cell‐free extracts, we selectively removed one of the isomers—occupying the right peak (retention time 9.0 min) of the doublet in the LC chromatogram (Figure 7A)—using a dehydrogenase assay with enzyme ThnO. Subsequently, the solvent was eliminated via vacuum evaporation and lyophilization. The dry sample was dissolved in 200 μL of a 10% (v/v) diisopropylethylamine (DIPEA) solution in dichloromethane (DCM). The remaining free carboxylic acid was derivatized with pentafluorobenzyl bromide (PFB‐Br) (Gors et al. 2022) by adding 200 μL of a 10% (v/v) PFB‐Br solution in DCM, incubating at 50°C for 1 h, and subsequently drying at 45°C. Afterwards, the thioester bond was broken under basic conditions in 600 μL of a phosphate‐buffered saline (PBS; components: 137 mM NaCl, 2.7 mM KCl, 8 mM Na_2_HPO_4_ and 2 mM KH_2_PO_4_) solution (CLS Cell Lines Service GmbH, Eppelheim, Germany), previously adjusted to pH 10 with sodium hydroxide (18.6 mM). Following an incubation period of 1 h at 30°C, 70 μL acetic acid (50%, v/v) was added to create slightly acidic conditions, followed by three times extraction with 200 μL hexane each. After evaporating the organic solvent, the derivatized and de‐esterified samples were dissolved in 100 μL MeOH and then subjected to GC–MS analysis. As a control, the CoA thioesters mixture without dehydrogenase treatment underwent the same preparation steps and GC–MS analysis.

GC–MS measurements were performed with an Agilent 8890 GC system coupled to an Agilent 7010B GC/MS Quadrupole mass spectrometer (Agilent Technologies, Santa Clara, USA). The injection was carried out by an Automated Liquid Sampler (Model: G4567A; Agilent Technologies). One microliter of sample was analysed with a 1:10 split injection and a ZB‐SemiVolatiles column (30 m × 0.25 mm, 0,25 μm; Phenomenex, Aschaffenburg, Germany). The temperature gradient was 0–1 min isothermal at 35°C as initial condition, 1–1.4 min linear to 50°C with 40°C/min, 1.4–55.4 min linear to 320°C with 5°C/min and 55.4–65.4 min isothermal at 320°C, followed by cooling down and a post run of 1 min at 50°C. Helium was used as the carrier gas with a constant flow rate of 1.3 mL/min. The mass spectrometer was equipped with a High‐Efficiency Source (Agilent Technologies). The positive ionisation was carried out at a source temperature of 230°C and an electron energy of 70 eV.

### 2.13 NMR Spectroscopy

To determine the double bond position of the product of the dehydrogenase reaction, the product peaks with mass of *m*/*z* = 934 from the enzyme assays with ThnO were collected by LC–MS. The structure elucidation of the purified compounds was performed by ^1^H NMR spectroscopy with an Avance II spectrometer (Bruker Biospin AG) operating at a frequency of 700.23 MHz and equipped with a helium‐cooled QXI cryoprobe. The sample was dissolved in D_2_O; the residual solvent signal was suppressed by applying excitation sculpting. D_2_O was used as secondary chemical shift reference (4.79 ppm); all spectra were recorded at 25°C. In the ROESY experiment, a 300 ms spinlock pulse was applied to detect the significant cross peaks. Processing was done using the Topspin 3.7 software (Bruker).

### 2.14 Sequence Alignment and Homology Modelling

Amino acid sequences of ThnO homologues were retrieved from SwissProt database using EBI BLAST server and were aligned using Clustal W (genome.jp/tools‐bin/clustalw). A neighbour‐joining tree was constructed based on the alignment using the MEGA11 software and Program iTOL (itol.embl.de). Amino acid sequences of CoA transferase homologues were mainly recorded in a previous publication (Hackmann 2022), and sequence alignment with ThnP was conducted using program MUSCLE in MEGA11 software (Edgar 2004).

The homology model of ThnO was performed using the Swiss‐Model Automated Comparative Protein Modelling Server (https://swissmodel.expasy.org/). FAD was transplanted into the ThnO active site with Alphafill (https://alphafill.eu/) and the substrate (1*R*,2*R*)‐2‐carboxycyclohexylacetyl‐CoA was docked into the active site using the Dockthor server (https://dockthor.lncc.br/v2/). The AlphaFold structure of ThnP was retrieved from the UniProt database. PyMOL software was used to visualise the structure model.

## 3 Results

### 3.1 Molecular Properties of the Acyl‐CoA Dehydrogenases ThnO and ThnT

In order to identify the acyl‐CoA dehydrogenase responsible for oxidising the carboxymethyl side chain of 2‐(carboxymethyl)cyclohexane‐1‐carboxylic acid coenzyme A thioester, ThnO and ThnT were expressed in *E. coli* as either His6‐tagged or Twin‐strep‐tagged proteins. However, only the Twin‐strep‐tagged enzymes showed activity and appeared yellow indicating a flavine cofactor. ThnO was purified to homogeneity with a protein content of 6.6 mg/mL (Figure S3). The purification of ThnT resulted in lower concentrations of 0.7 mg/mL and the protein was not purified to homogeneity. The predicted monomer molecular mass of ThnO and ThnT with Twin‐strep tag were 44.8 and 46.0 kDa, respectively, which agreed with their migration in SDS‐gels. Blue‐native PAGE gel electrophoresis of ThnO exhibited a molecular weight of approximately 157 kDa when compared to protein standards. The native molecular weight of ThnO as estimated by size exclusion chromatography was 168 kDa (Figure S3), suggesting a homotetrameric composition for ThnO, which is typical for acyl‐CoA dehydrogenases (Kim and Miura 2004).

ThnO had a UV–vis flavin spectrum with spectral maxima at 380 and 450 nm indicating oxidised flavin (Figure S4). The absorption peaks for flavin vanished after reduction with 80 μM sodium dithionite. The flavin cofactor was extracted from ThnO by denaturation with methanol and following mass spectrometric analysis revealed a metabolite with *m*/*z* = 786 assigned to the protonated molecular ion [M + H]^+^ with identical retention time to an FAD standard, confirming the identity of the cofactor as FAD. Quantification of the FAD content per protein monomer was performed by LC–MS determination of the flavin and revealed 3.2 FAD/tetramer (0.8 FAD per 44.8‐kDa monomer) indicating 1 FAD per protein subunit.

### 3.2 2‐(Carboxymethyl)cyclohexane‐1‐Carboxylic Acid CoA Thioesters Conversion With Recombinant Enzymes

**FIGURE 2:**
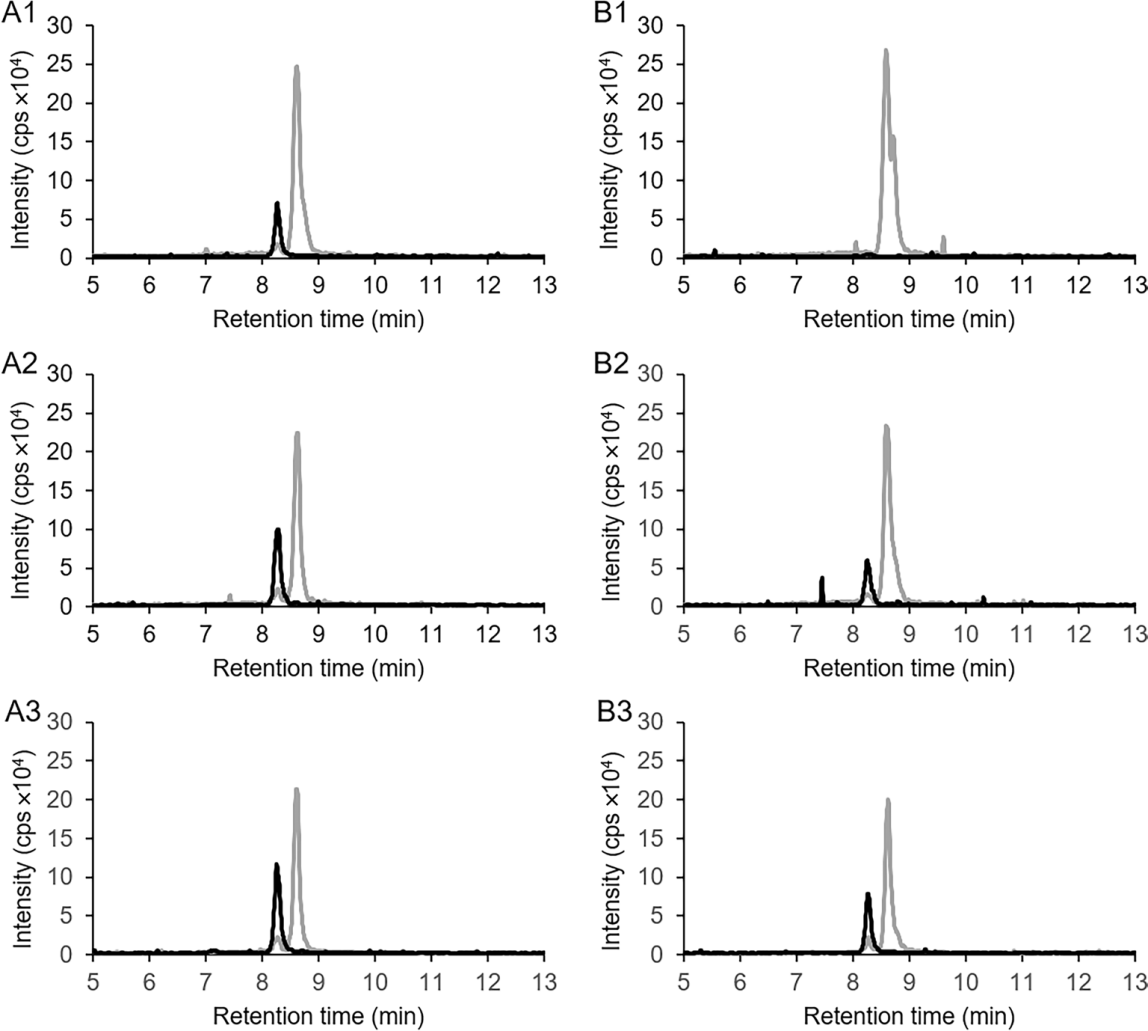
LC–MS chromatograms showing the conversion of substrate 2‐(carboxymethyl)cyclohexane‐1‐carboxylic acid CoA thioesters produced in cell‐free extracts (mixture of compounds 16 and 17 in Figure 4) by the heterologously expressed dehydrogenases ThnO (A) and ThnT (B). A1: Conversion of substrate with ThnO, *t* = 0 min. A2: *t* = 15 min. A3: *t* = 45 min. B1: Conversion of substrate with ThnT, *t* = 0 min. B2: *t* = 15 min. B3: *t* = 45 min. Grey lines: Ion counts for 2‐(carboxymethyl)cyclohexane‐1‐carboxylic acid CoA thioesters with *m*/*z* = 936. Black lines: Ion counts of the dehydrogenase product 2‐carboxycyclohexylideneacetyl‐CoA with *m*/*z* = 934. 23 μg of ThnO was used in panel A compared to 53 μg of ThnT in Panel B indicating that ThnO is more active than ThnT.

To investigate the activity of the two acyl‐CoA dehydrogenase candidates, the natural substrate was synthesised biologically with cell‐free extracts of strain N47. THN‐CoA was converted to CoA thioesters of 2‐(carboxymethyl)cyclohexane‐1‐carboxylic acid with N47 cell free extract by using NADPH as electron donor of THN‐CoA reductase and CoA‐SH as CoA donor for the downstream β‐oxoacyl‐CoA thiolase (Figure 1B). CoA thioesters of 2‐(carboxymethyl)cyclohexane‐1‐carboxylic acid accumulated because of a lack of electron acceptor for the downstream dehydrogenase. This natural intermediate appeared as two partly overlapping peaks in the LC–MS chromatogram eluting around 8.6 and 8.8 min (Figure 2B), which most likely represented two isomers with the same mass of *m*/*z* = 936 but different positions of the CoA thioester at either the carboxyl‐ or the carboxymethyl‐residue.

After adding 23 μg of the purified recombinant acyl‐CoA dehydrogenase ThnO to the produced compound, the right peak (retention time 8.7 min) of 2‐(carboxymethyl)cyclohexane‐1‐carboxylic acid CoA thioesters diminished completely within 15 min, whereas the left peak (retention time 8.6 min) decreased only slightly (Figure 2A), resulting in the conversion of 40% of the substrate (0.16 mM out of 0.4 mM) within 15 min. A new compound with *m*/*z* = 934 appeared at 8.3 min elution time, indicating an oxidised product of the acyl‐CoA dehydrogenase reaction, most likely 2‐carboxycyclohexylideneacetyl‐CoA (compound 9 in Figure 1B).

Similar results were observed with 53 μg purified recombinant enzyme ThnT (approximately 18 μg of actual ThnT protein based on a purity of 34%) (Figure 2B), but with a 41‐fold lower specific activity compared to ThnO and a substrate conversion of 23% (0.09 mM out of 0.4 mM) within 15 min, indicating that ThnO is the specific dehydrogenase for the oxidation of the CoA thioesters of 2‐(carboxymethyl)cyclohexane‐1‐carboxylic acid and not ThnT. This difference in activity is based on an accurate estimation of the actual amount of ThnT with the ImageJ software, which quantifies impurities in SDS‐gel, ensuring that the comparison reflects the true catalytic capacity of the enzymes.

The demonstrated enzyme activities of the gene products ThnO and ThnT also proved that the *thn* operon (Figure 1A) encodes the downstream degradation pathway of anaerobic naphthalene degradation supporting earlier proteogenomic work (Bergmann, Selesi, and Meckenstock 2011).

### 3.3 Identification of Substrate and Product of the Acyl‐CoA Dehydrogenases ThnO and ThnT

**FIGURE 3:**
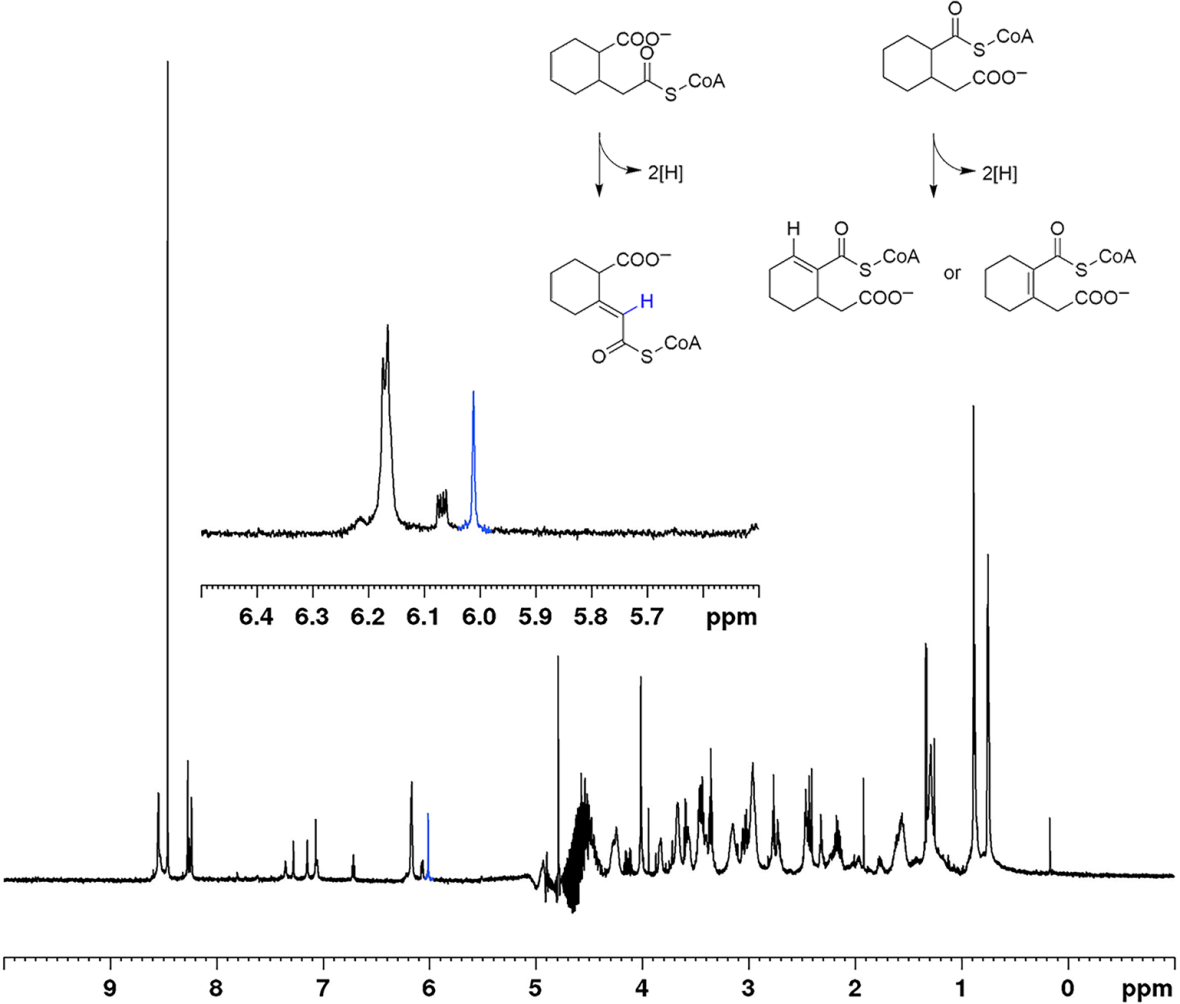
^1^H NMR spectra of the product of dehydrogenase ThnO with *m*/*z* = 934 (700.23 MHz, 298 K, D_2_O), as well as the three possible reactions catalysed by ThnO. The relevant olefinic proton of the double bond introduced by the dehydrogenase reaction is marked in blue.

Theoretically, the dehydrogenase reaction catalysed by ThnO/ThnT could take place at the acetyl‐CoA side chain of the 2‐(carboxymethyl)cyclohexane‐1‐carboxylic acid CoA thioesters or at the carboxyl‐CoA with a dehydrogenation in the ring (Figure 3). In order to identify the location of the double bond in the dehydrogenase product, we collected the product peaks with mass of *m*/*z* = 934 from the LC–MS analyses of enzyme assays with ThnO and performed ^1^H NMR spectroscopy. The ^1^H NMR spectrum showed resonances of the cyclohexane ring overlapping with those of the CoA at range of 4.5–1 ppm. The relevant olefinic proton of the double bond introduced by the dehydrogenase reaction was indicated by a single peak at 6.01 ppm (Figure 3). The identification was made by a ROESY experiment, which unambiguously showed a dipolar interaction to protons of the cyclohexane ring. If the double bond were located in the cyclohexane ring, a splitting of this peak (into a doublet of doublet) would be expected due to spin–spin coupling with the next CH_2_ protons within the ring. Only when the double bond was introduced in the carboxymethyl‐residue the coupling of the olefinic proton with CH_2_ protons could not be observed, because the β‐carbon atom of the acetyl‐side chain is a tertiary carbon atom, which does not have an olefinic proton after the dehydrogenase reaction. Consequently, the substrate of the acyl‐CoA dehydrogenases ThnO must be 2‐carboxycyclohexylacetyl‐CoA, which was oxidised via introduction of a C=C double‐bond in α,β‐position of the carboxymethyl‐residue to the product 2‐carboxycyclohexylideneacetyl‐CoA. Given that the product of the reaction catalysed by ThnT exhibited the same retention time in LC–MS analysis as that of ThnO, we conclude that both the substrate and product of ThnT are identical to those of ThnO.

### 3.4 Identification of the Substrate Isomer

**FIGURE 4:**
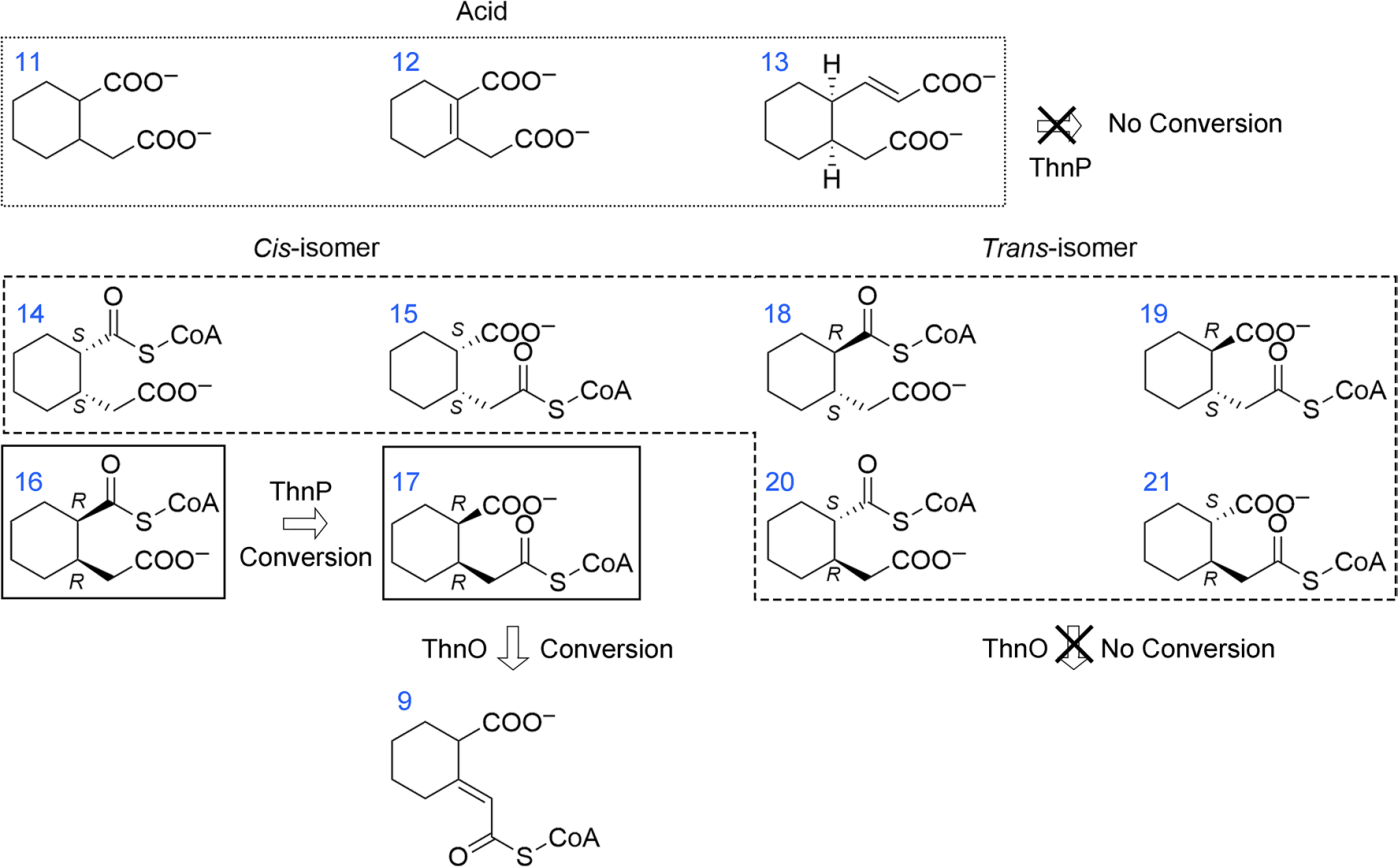
Isomers of 2‐(carboxymethyl)cyclohexane‐1‐carboxylic acid CoA thioesters (compound 14 to 21) and *cis*‐2‐(carboxymethyl)cyclohexane‐1‐carboxylic acid (11) as well as the structural analogs 2‐(carboxymethyl)cyclohex‐1‐enecarboxylic acid (12) and *cis*‐(E)‐3‐(2‐(carboxymethyl)cyclohexyl) acrylic acid (13) applied in enzyme assays with heterologously produced and purified enzymes ThnO and ThnP. Substrates in the solid frames are the real substrates of acyl‐CoA dehydrogenase ThnO and CoA transferase ThnP; whereas substrates in the dashed frame cannot be converted by ThnO and substrates in the dotted border cannot be converted by ThnP. Substrates in the dotted frame are free acids which were tested for intermolecular CoA transfer of ThnP with succinyl‐CoA and acetyl‐CoA.

In order to elucidate which isomers are converted by the acyl‐CoA dehydrogenases, acyl‐CoA thioesters of *cis*‐isomers (1*R*,2*R*/1*S*,2*S*) and *trans*‐isomers (1*R*,2*S*/1*S*,2*R*) of 2‐(carboxymethyl)cyclohexane‐1‐carboxylic acid were chemically synthesised via carbonyldiimidazole activation and used for enzyme assays. Considering that in the synthesis of the CoA thioesters the reaction can either lead to a CoA thioester at the carboxyl‐ or at the carboxymethyl‐residue of the 2‐(carboxymethyl)cyclohexane‐1‐carboxylic acid, the racemic *cis*‐/*trans*‐isomer theoretically formed eight CoA thioesters with the same mass of *m*/*z* = 936 (Figure 4). It was unlikely that carbonyldiimidazole simultaneously attacked two carboxyl residues because a di‐CoA thioester with *m*/*z* = 1685 was not detected in LC–MS (data not shown).

**FIGURE 5:**
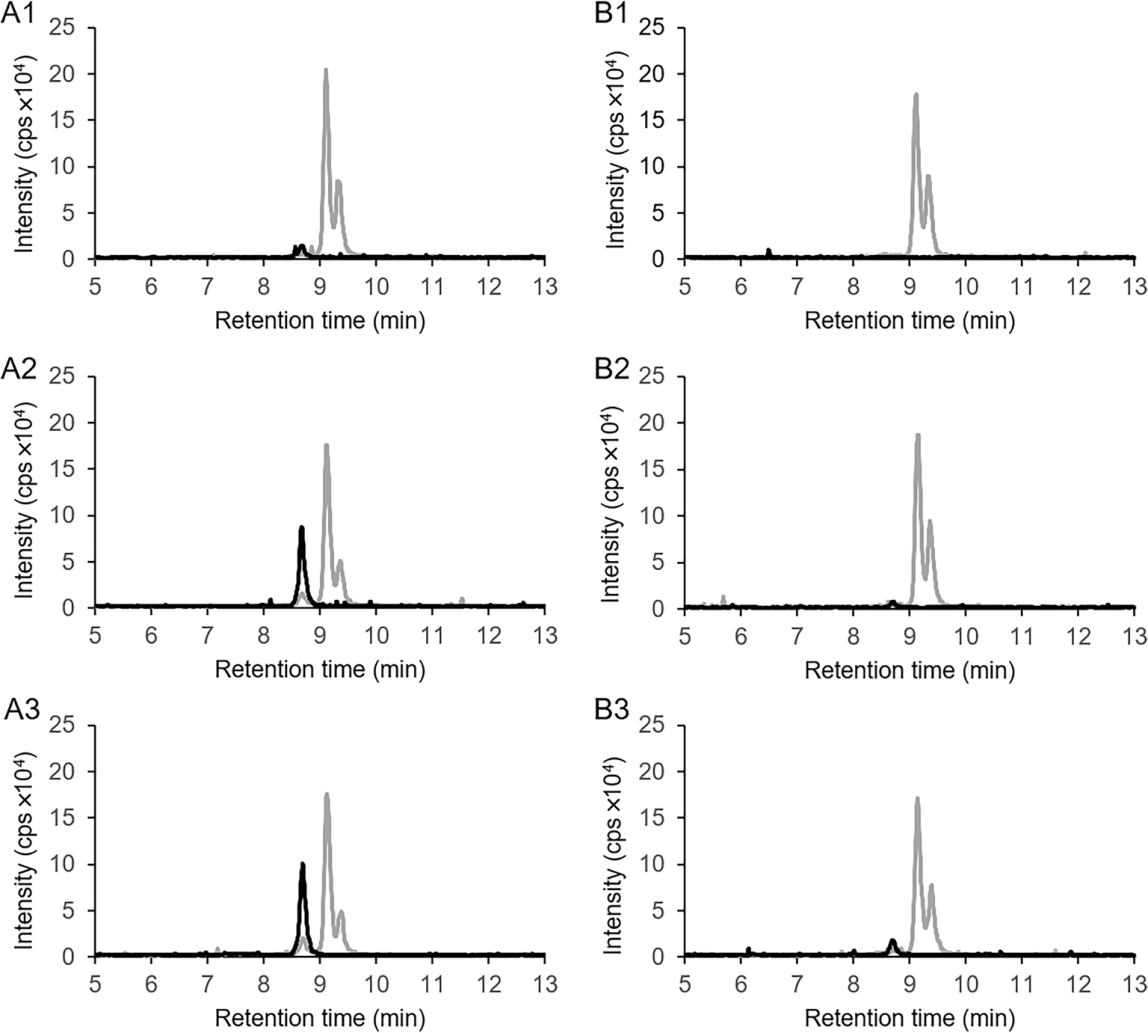
LC–MS chromatograms showing the conversion of substrate CoA thioesters of chemically synthesised *cis*‐2‐(carboxymethyl)cyclohexane‐1‐carboxylic acid (mixture of compounds 14–17 in Figure 4) by heterologously expressed ThnO (A) and ThnT (B) at the same protein concentration of 21.2 μg/mL. A1: Conversion of the substrate with ThnO at *t* = 0 min. A2: *t* = 7 min. A3: *t* = 30 min. B1: Conversion of substrate with ThnT, *t* = 0 min. B2: *t* = 7 min. B3: *t* = 30 min. Grey lines: Ion counts for *cis*‐2‐(carboxymethyl)cyclohexane‐1‐carboxylic acid CoA thioesters with *m*/*z* = 936. Black lines: Ion counts of conversion product 2‐carboxycyclohexylideneacetyl‐CoA with *m*/*z* = 934.

The four different *cis*‐CoA thioester isomers showed two overlapping peaks in the LC–MS chromatogram (Figure 5), which implies CoA thioester formation at either of the two possible carboxyl groups. In contrast to previous results, where 2‐(carboxymethyl)cyclohexane‐1‐carboxylic acid CoA thioesters was produced in cell free extract, only 47% of the right peak (retention time 9.4 min) of the chemically synthesised *cis*‐isomers (mix of compounds 14–17 in Figure 4) were degraded by 4 μg ThnO within 15 min (Figure 5A). This degradation corresponded to a conversion of 0.03 mM out of a total of 0.2 mM substrate, yielding a specific activity of 0.24 μmol min^−1^ mg^−1^ protein. The peak area of the left peak (retention time 9.1 min) simultaneously decreased to a slight extent, probably due to the overlap with the right peak. The *trans*‐isomers ((1*R*,2*S*) and (1*S*,2*R*)) could neither be converted by ThnO nor ThnT (data not shown) suggesting that the two dehydrogenases are specific to either the (1*R*,2*R*) or (1*S*,2*S*) *cis*‐isomer. With equivalent protein quantities, the non‐specific ThnT catalysed substrate conversion at only 0.016 μmol min^−1^ mg^−1^ protein (Figure 5B), with just 13% of the right peak (retention time 9.4 min) degraded within 30 min, corresponding to 0.01 mM conversion out of a total of 0.2 mM substrate. These results suggest ThnT lacks specificity for this reaction. Hence, we conclude that ThnO is the 2‐carboxycyclohexylacetyl‐CoA dehydrogenase catalysing the oxidation of the *cis* (1*R*,2*R*) or (1*S*,2*S*) isomer of 2‐carboxycyclohexylacetyl‐CoA to 2‐carboxycyclohexylideneacetyl‐CoA.

To acquire the enantiomerically pure substrate we conducted a derivatization with enantiopure alcohol (*R*)‐2‐phenyl‐1‐propanol to form a mixture of diastereomeric diesters with (*S*,*S*,*R*,*R*) and (*R*,*R*,*R*,*R*) configuration (compounds 25 and 26 in Figure S1) (see Figure S1 for reaction scheme). Separation of these diastereomers was possible by chiral stationary phase HPLC. As expected, both diastereomers were present in approximately equal amounts (Figure S2). After separation, the diastereomerically pure esters were hydrolysed under mild conditions to avoid epimerization to the unwanted *trans*‐dicarboxylic acids. The ^1^H‐NMR spectra of the isolated acids after hydrolysis were identical to that of the racemate (Figure S5), indicating that no epimerization took place during hydrolysis. The physical data and NMR spectra of compounds involved in the preparation of the enantiomerically pure substrate are provided in Appendix S1. In that way, both pure enantiomers of *cis*‐2‐(carboxymethyl)cyclohexane‐1‐carboxylic acid could be obtained and further functionalised as the corresponding CoA thioesters. The activity of ThnO using the enantiomerically pure CoA thioesters revealed a 340‐fold higher catalytic activity exclusively towards one of the substrates, which was identified as the CoA thioesters of (1*R*,2*R*)‐2‐(carboxymethyl)cyclohexane‐1‐carboxylic acid afterwards, demonstrating its stereospecific discrimination between enantiomers.

The IR and VCD spectra of the non‐CoA derivative of the real substrate in CDCl_3_ were compared with the computed pattern of monomeric species and no good match was found. Hence, as dimerization in non‐polar solvents is very likely, spectra of the dimeric species were computed and compared to the experiment. Analysis of the spectral signatures suggested that the real substrate possesses a (*R*,*R*)‐configuration. A closer look at the dimeric structures revealed that both open chain dimers as well as “folded” structures like the minimum energy dimer could form (Figure S6B,C). In order to further confirm the configuration, we added two equivalents of 7‐azaindole (7AI) to the solution of the non‐CoA derivative of the real substrate in order to break dimers (Grassin, Santoro, and Merten 2022). Expectedly, the calculated spectra simulated for (7AI)_2−_complexes also matched well with the experimental pattern (Figure S6A), so that we can unambiguously assign the stereochemistry as (1*R*,2*R*)‐2‐(carboxymethyl)cyclohexane‐1‐carboxylic acid.

**FIGURE 6:**
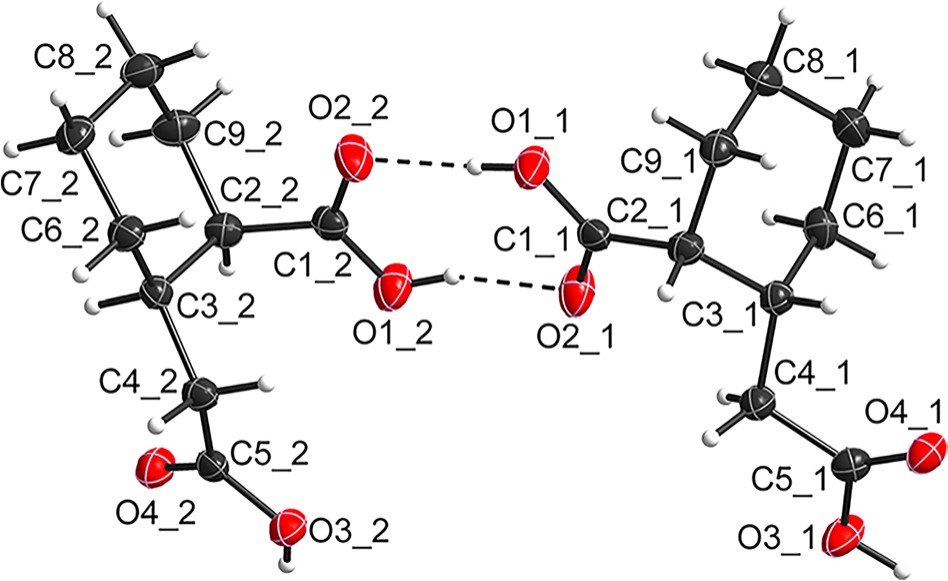
ORTEP‐representation of the asymmetric unit of enantiopure non‐CoA derivative of the real substrate of ThnO ((1*R*,2*R*)‐2‐(carboxymethyl)cyclohexane‐1‐carboxylic acid) in the solid state. The alternate positions of the hydrogen atoms were omitted for clarity. The displacement ellipsoids are drawn at 50% probability levels. Hydrogen atoms are displayed as spheres of arbitrary radii.

The configuration of the non‐CoA derivative of the real substrate was also established by single‐crystal X‐ray structure determination. The ORTEP representations of the molecular structure are given in Figure 6, which confirm the absolute chirality of stereo centers as (1*R*,2*R*), consistent with the results of the VCD spectra. Additional details of the crystal structure refinement are provided in Table S3 and in the Crystallographic Information File (CIF) deposited with the Cambridge Crystallographic Data Centre (CCDC‐2369957).

Determination of apparent kinetic parameters of ThnO revealed apparent Km and Vmax values of 61.5 ± 10.3 μM and 1.2 ± 0.1 μmol min^−1^ mg^−1^ protein (Table 1 and Figure S7). The observed absence of substrate‐inhibited Michaelis–Menten enzyme kinetics on ThnO suggests no inhibition from the “wrong” enantiomer in racemic mixtures. Consistently, enantiomerically mixed substrate did not diminish substrate utilisation efficiency compared to assays with enantiomerically pure (1*R*,2*R*)‐isomer (data not shown), demonstrating that ThnO is able to efficiently discriminate against the substrate with the wrong conformation.

**TABLE 1:** Properties of acyl‐CoA dehydrogenase ThnO and ThnT heterologously expressed in *Escherichia coli*.

Property	Profile for enzyme	
	ThnO	ThnT
Native molecular mass (kDa)^a^	44.8	46.0
Subunit composition	*α*_4_	—^b^
Gene	N47_E41350	N47_E41310
FAD content per subunit	0.8	—^b^
*V*_max_ (μmol min^−1^ mg^−1^)	1.2 ± 0.1	—^b^
*K*_m_ (μM)	61.5 ± 10.3	—^b^

^a^ Molecular mass including Twin‐strep tag.

^b^ Not measured because of inhomogeneity of ThnT.

### 3.5 ThnP Is an Internal CoA Transferase of 2‐(Carboxymethyl)cyclohexane‐1‐Carboxylic Acid

**FIGURE 7:**
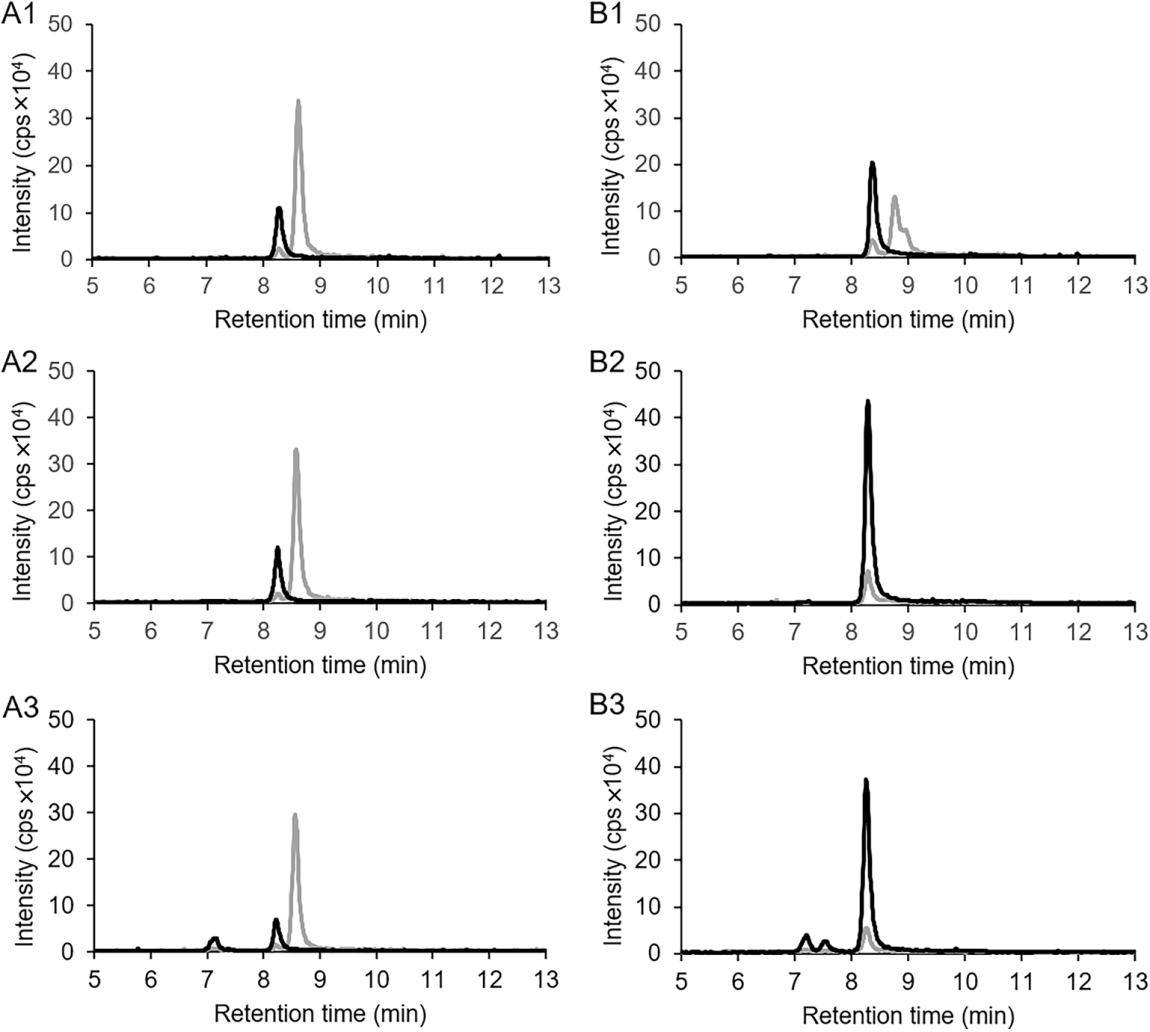
LC–MS chromatograms showing the conversion of substrate 2‐(carboxymethyl)cyclohexane‐1‐carboxylic acid CoA thioesters produced with cell free extract of culture N47 (mixture of compounds 16 and 17 in Figure 4), by either heterologously expressed ThnO (A) or a mixture of ThnO and ThnP (B). A1: Conversion of substrate with ThnO, *t* = 0 min. A2: *t* = 5 min. A3: *t* = 60 min. B1: Conversion of substrate with ThnO and ThnP, *t* = 0 min. B2: *t* = 5 min. B3: *t* = 60 min. Grey lines: Ion counts for 2‐(carboxymethyl)cyclohexane‐1‐carboxylic acid CoA thioesters with *m*/*z* = 936. Black lines: Ion counts of dehydrogenase product 2‐carboxycyclohexylideneacetyl‐CoA with *m*/*z* = 934.

To investigate the conversion of 2‐(carboxymethyl)cyclohexane‐1‐carboxyl‐CoA, the putative CoA transferase gene *thnP* was cloned and expressed in *E. coli* as a His‐tagged protein (46.5 kDa, Figure S3). There was no reaction observable when the heterologously expressed ThnP alone was added to the cell free extract‐derived or the chemically synthesised CoA thioesters of 2‐(carboxymethyl)cyclohexane‐1‐carboxylic acid with *m*/*z* = 936 (data not shown). However, adding both ThnP and the dehydrogenase ThnO (24 μg and 112 μg respectively) to the enzyme assay allowed for complete conversion of the 2‐(carboxymethyl)cyclohexane‐1‐carboxylic acid CoA thioesters mix produced with cell‐free extract (Figure 7B2). In contrast, when 24 μg ThnO alone was added, only one of the two regioisomers was converted (see above and Figure 7A2), with 37% of the substrate (12 μM out of 32 μM) undergoing transformation within 5 min.

We interpret these results as such that ThnP is a CoA‐transferase that catalyses an intramolecular CoA‐transfer between the carboxyl‐ and the carboxymethyl‐residue of 2‐(carboxymethyl)cyclohexane‐1‐carboxylic acid CoA thioester. Without the CoA‐transferase ThnP, the dehydrogenase ThnO can only oxidise the 2‐(carboxycyclohexyl)acetyl‐CoA. This leads to the decrease of only the right peak (retention time 9.0 min) of the 2‐(carboxymethyl)cyclohexane‐1‐carboxylic acid CoA thioesters in (Figure 7A). The addition of the CoA‐transferase ThnP produced a chemical equilibrium between 2‐carboxycyclohexylacetyl‐CoA and 2‐(carboxymethyl)cyclohexane‐1‐carboxyl‐CoA, which means that with depletion of 2‐carboxycyclohexylacetyl‐CoA by the ThnO dehydrogenase activity, the transferase reaction regenerated 2‐carboxycyclohexylacetyl‐CoA by intramolecular CoA‐transfer from 2‐(carboxymethyl)cyclohexane‐1‐carboxyl‐CoA until both compounds were fully depleted (Figure 1B).

To confirm the assignment of the two substrate peaks to isomers with distinct side chains, we selectively removed one of the substrates using a dehydrogenase assay (Figure 7A), followed by derivatization and GC–MS analysis. However, we could not distinguish the two isomers because no structure‐specific fragment patterns or signals could be attributed to the analytes (Figure S8).

**FIGURE 8:**
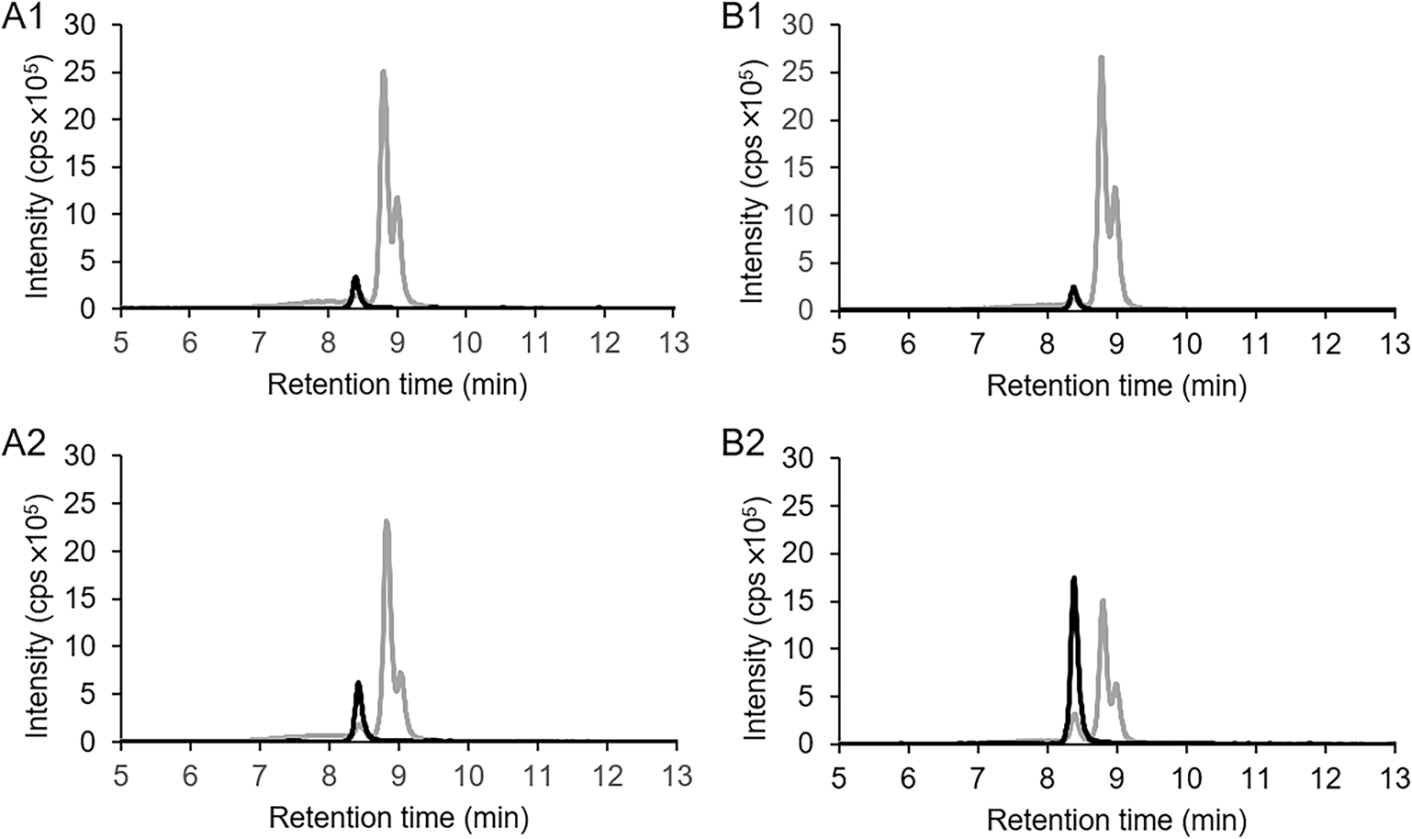
LC–MS chromatograms showing the conversion of substrate CoA thioesters of chemically synthesised *cis*‐2‐(carboxymethyl)cyclohexane‐1‐carboxylic acid (mixture of compounds 14–17 in Figure 4) by either the heterologously expressed enzymes dehydrogenase ThnO (A) or a mixture of ThnO and the CoA‐transferase ThnP (B). A1: Conversion of substrate with ThnO, *t* = 0 min. A2: *t* = 15 min. B1: Conversion of substrate with ThnO and ThnP, *t* = 0 min. B2: *t* = 15 min. Grey lines: Ion counts for *cis*‐2‐(carboxymethyl)cyclohexane‐1‐carboxylic acid CoA thioesters with *m*/*z* = 936. Black lines: Ion counts of the dehydrogenase product 2‐carboxycyclohexylideneacetyl‐CoA with *m*/*z* = 934.

The results observed using cell free extract‐derived substrate were in agreement with the results of enzyme assays with the chemically synthesised *cis*‐isomers of 2‐(carboxymethyl)cyclohexane‐1‐carboxylic acid CoA thioesters (mix of compounds 14–17 in Figure 4), where significantly more substrate was converted when both ThnP and ThnO (both 9 μg) were added (Figure 8B2). Specifically, 42% of the substrate (0.08 mM out of 0.2 mM) was converted within 15 min, compared with 21% (0.04 mM out of 0.2 mM) when only 9 μg of ThnO was added (Figure 8A2). Only approximately half of the chemically synthesised *cis*‐2‐(carboxymethyl)cyclohexane‐1‐carboxylic acid CoA thioesters underwent degradation by the combination of ThnO and ThnP due to equal amounts of (1*R*,2*R*)‐ and (1*S*,2*S*)‐2‐(carboxymethyl)cyclohexane‐1‐carboxylic acid present. Given ThnO's specific degradation of (1*R*,2*R*)‐2‐carboxycyclohexylacetyl‐CoA as stated before, ThnP was expected to convert only (1*R*,2*R*)‐2‐(carboxymethyl)cyclohexane‐1‐carboxyl‐CoA for subsequent dehydrogenase reactions. The remaining (1*S*,2*S*)‐2‐(carboxymethyl)cyclohexane‐1‐carboxyl‐CoA and (1*S*,2*S*) carboxycyclohexylacetyl‐CoA was still in equilibrium, leaving uncertainty regarding ThnP's ability to transform (1*S*,2*S*) stereoisomers.

Besides the product of the dehydrogenase reaction, 2‐carboxycyclohexylideneacetyl‐CoA with *m*/*z* = 934, which eluted at 8.3 min in the LC–MS chromatogram (Figure 7A2), a new product with the same *m*/*z* = 934 eluted at 7.2 min (Figure 7A3), after prolonged incubation of 1 h, consistent with the result in Figure 2A. The new product at 7.2 min appeared only when high amounts of ThnO were used and is a so far unknown by‐product of the dehydrogenase reaction. When ThnP was added to these assays, another new product with *m*/*z* = 934 was found that eluted at 7.5 min (Figure 7B3) and is most likely just a CoA‐transferase product of the by‐product of the dehydrogenase reaction.

No CoA transferase activity was observed with either *cis*‐2‐(carboxymethyl)cyclohexane‐1‐carboxylic acid itself or several structure analogs such as 2‐(carboxymethyl)cyclohex‐1‐enecarboxylic acid and *cis*‐(E)‐3‐(2‐(carboxymethyl)cyclohexyl) acrylic acid (Figure 4) serving as CoA acceptors when acetyl‐CoA or succinyl‐CoA were used as CoA donors, indicating ThnP as a bona fide intramolecular CoA transferase.

### 3.6 2‐(Carboxymethyl)cyclohexane‐1‐Carboxylic Acid CoA Thioesters Conversion by Cell Free Extract

Cell free extract of naphthalene‐grown cells consumed *cis*‐2‐(carboxymethyl)cyclohexane‐1‐carboxylic acid CoA thioesters at a rate of 4.6 nmol min^−1^ mg^−1^ protein, which was observed by monitoring the conversion of *cis*‐2‐(carboxymethyl)cyclohexane‐1‐carboxylic acid CoA thioesters with *m*/*z* 936 to 2‐(1‐hydroxy‐2‐carboxycyclohexyl)acetyl‐CoA with *m*/*z* 952 by a combination of CoA‐transferase, acyl‐CoA dehydrogenase and subsequent enoyl‐CoA hydratase (Figures S9B and 1B). The product of the dehydrogenase reaction with *m*/*z* = 934 did not accumulate, because the downstream enoyl‐CoA hydratase present in the cell free extract was highly active (data not shown), depleting the 2‐carboxycyclohexylideneacetyl‐CoA with *m*/*z* = 934. Similar to the in vitro conversion with a combination of heterologously expressed ThnO and ThnP, only 39% of the *cis*‐2‐(carboxymethyl)cyclohexane‐1‐carboxylic acid CoA thioesters was converted by cell free extracts within 5 min, corresponding to 0.08 mM out of 0.2 mM substrate. This conversion was limited because the natural acyl‐CoA dehydrogenase and CoA‐transferase in N47 cell free extract could not degrade the (1*S*,2*S*)‐2‐(carboxymethyl)cyclohexane‐1‐carboxylic acid CoA thioesters.

### 3.7 Sequence Alignment

The sequence distance relationship of ThnO/ThnT was analysed with selected members from other acyl‐CoA dehydrogenase subclasses, showing that ThnO shares 45% identity with glutaryl‐CoA dehydrogenase from the sulfate‐reducing bacterium *Desulfococcus multivorans* (Figure S10A). Multiple sequence alignment indicated that the catalytic glutamate (E363) of ThnO is located in the carboxy‐terminal loop JK that is presumably involved in initiating the reaction by abstracting the proton at the C2 atom of the substrate 2‐carboxycyclohexylacetyl‐CoA (Figure S10B). The Glu residue is conserved in most FAD‐dependent dehydrogenases, apart from long‐chain acyl‐CoA dehydrogenases, isovaleryl‐CoA dehydrogenase and benzylmalonyl‐CoA dehydrogenase, which have glycine or alanine instead at the corresponding position (Djordjevic et al. 1994; Kim and Miura 2004; Schühle et al. 2021).

The strictly conserved amino acid regions S/TEP/A*XX*GS and K*X*W/FIT/S involved in the binding of FAD were also identified in ThnT (Figure S10B) (Schürmann et al. 2015). However, the conserved S/TEP/A*XX*GS motif is substituted by TEPNAAT in ThnO. A similar change can be found in the butyryl‐CoA dehydrogenase of the fermentative obligate anaerobe *Clostridium acetobutylicum*. Amino acids responsible for positioning the CoA moiety in acyl‐CoA dehydrogenases of known structures (Kim, Wang, and Paschke 1993; Erb, Fuchs, and Alber 2009) are also conserved in ThnO (R243, F271)/ThnT (R249, F277) (Figure S10B).

**FIGURE 9:**
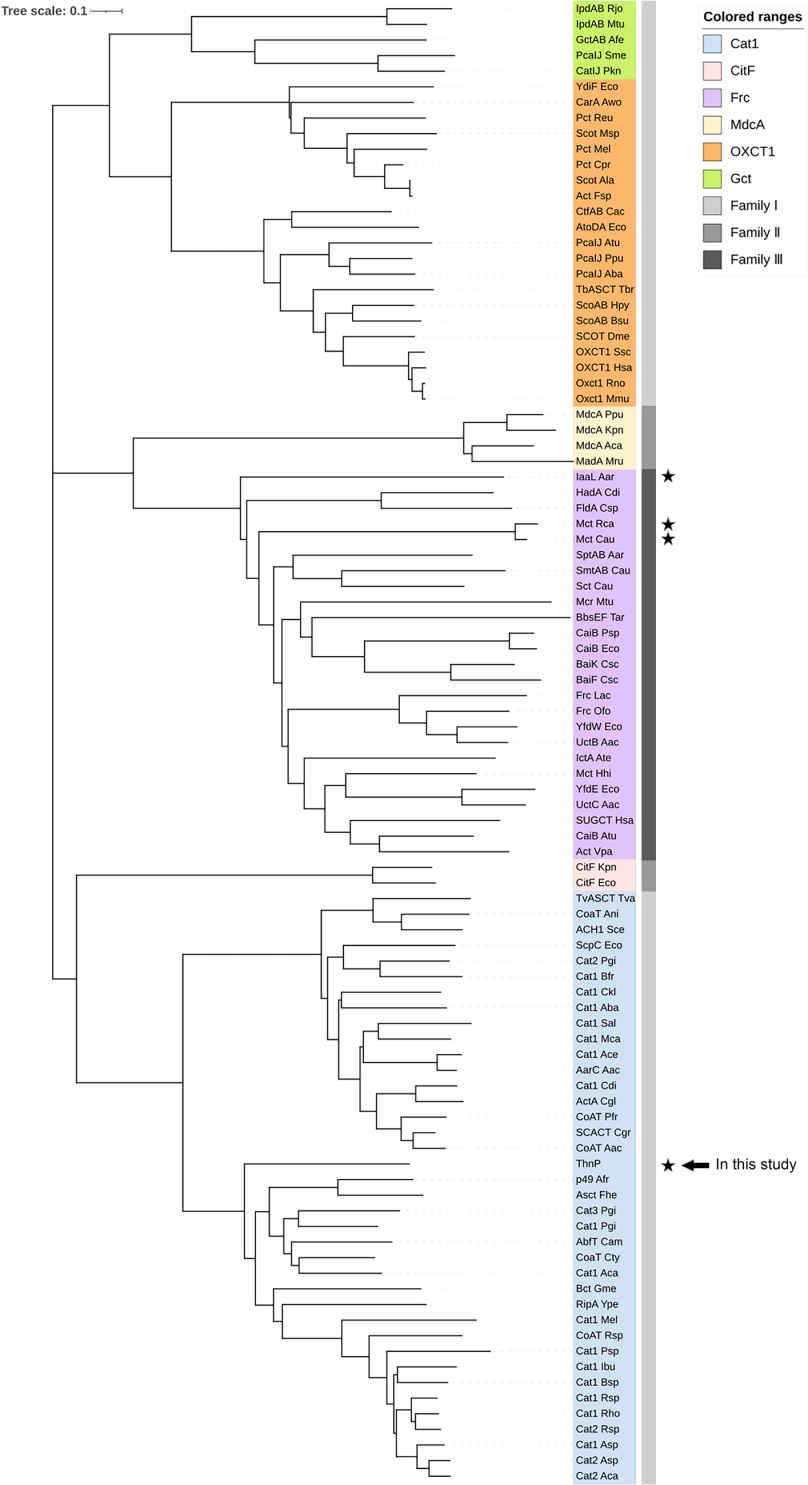
Modified neighbour‐joining tree of CoA transferase ThnP homologous composed of sequences from studies of Hackmann (2022) or Min et al. (2022) showing the classification of CoA‐transferase by Heider (2001) with grey code and Hackmann (2022) with vibrant colour code. The enzyme ThnP involved in this study is marked with an arrow. Enzymes with intramolecular CoA transfer activity are marked with stars. IaaL (phenylsuccinyl‐CoA transferase) from *A. aromaticum* exhibits both intermolecular and intramolecular CoA transfer activity, whereas Mct (mesaconyl‐CoA C1‐C4 CoA transferase) from *C. aurantiacus* and *R. castenholzii* only catalyse intramolecular transfer activity.

Phylogenetic analysis of ThnP indicated that it belongs to the class I CoA transferase family according to the most enduring classification model (Heider 2001). Furthermore, ThnP falls into the Cat1‐like subgroup in the optimised phylogenetic trees of CoA transferases (Figure 9) (Hackmann 2022). The highest amino acid sequence identity (46%) was identified with butyryl‐CoA: acetate CoA transferase, which belongs to Cat1 subgroup and is involved in the butyrate synthesis pathway in the anaerobic bacterium *Porphyromonas gingivalis*. The high rate of amino acid sequence identities (24%–46%) with Cat1‐like CoA transferase suggests a similar ping‐pong mechanism, which involves the formation of an enzyme‐bound acyl‐glutamyl anhydride caused by the nucleophilic attack of the active site glutamic acid (Selmer and Buckel 1999). However, the conserved active glutamate of class I CoA transferase is replaced by G238 in the corresponding position in ThnP (Figure S11), suggesting a possible alternative catalytic mechanism.

## 4 Discussion

### 4.1 Confirmation of Thn Operon Function

Anaerobic degradation of naphthalene proceeds by activation through carboxylation, CoA‐ligation and successive ring reductions. After the first ring cleavage, the downstream pathway of anaerobic naphthalene degradation continues via β‐oxidation‐like reactions (Figure 1B) (Weyrauch et al. 2017).

The genes encoding THN‐CoA reductases are surrounded by a cluster of genes that encode hydratases, dehydrogenases, hydrolases, CoA‐transferases and thiolases (Selesi et al. 2010; Estelmann et al. 2015) with predicted functions fitting an involvement in the downstream pathway of anaerobic naphthalene degradation (Figure 1A and Table S1). PCR amplification‐based operon mapping showed that all 22 genes within this cluster are co‐transcribed during growth with naphthalene (Weyrauch 2016). In this study, we verified that the *thn* gene cluster encodes for the downstream pathway of anaerobic naphthalene degradation and we identified and described the acyl‐CoA dehydrogenases ThnO and ThnT as well as the internal CoA‐transferase ThnP.

### 4.2 Properties of Acyl‐CoA Dehydrogenase

Earlier conversion studies of CoA thioesters of *cis*‐2‐(carboxymethyl)cyclohexane‐1‐carboxylic acid in cell free extract of N47 suggested the involvement of an acyl‐CoA dehydrogenase (Weyrauch et al. 2017). In this study, we identified the corresponding acyl‐CoA dehydrogenase ThnO by heterologous expression and characterisation of purified proteins. A second enzyme with the same function, ThnT, showed only poor activity compared to ThnO and was hypothesized to be a promiscuous acyl‐CoA dehydrogenase responsible for a different reaction in the degradation pathway. Therefore, enzyme ThnT was not further analysed in detail because its physiological function is unclear, yet.

ThnO had a homotetrameric quaternary structure with one FAD molecule per subunit, consistent with most of the other members of the acyl‐CoA dehydrogenase superfamily (Kim and Miura 2004).

The NMR‐based identification of 2‐carboxycyclohexylideneacetyl‐CoA (Figure 3) as the oxidised product of 2‐carboxycyclohexylacetyl‐CoA conversion catalysed by ThnO (Figures 2A and 5A) proved that the dehydrogenation took place at the secondary C2 (α) and tertiary C3 (β) atom of the acetyl‐side chain. Sequence alignment with other flavin‐dependent acyl‐CoA dehydrogenases indicated a 41% identity between ThnO and iso(3)valeryl‐CoA dehydrogenase participating in leucine metabolism (Figure S10A) (Tiffany et al. 1997). Both 2‐carboxycyclohexylacetyl‐CoA and iso(3)valeryl‐CoA feature an uncommon tertiary β‐carbon atom from which the sole hydride is thought to be transferred to the isoalloxazine ring of FAD (Kim and Miura 2004).

The Vmax of recombinant ThnO (1.2 μmol min^−1^ mg^−1^ protein) and the specific activity of native acyl‐CoA dehydrogenase in N47 cell free extract (4.6 nmol min^−1^ mg^−1^) are relatively low compared to acyl‐CoA dehydrogenases in other organisms, such as the glutaryl‐CoA dehydrogenase from *Geobacter metallireducens* (3.2 μmol min^−1^ mg^−1^) or *Desulfococcus multivorans* (11 μmol min^−1^ mg^−1^), cyclohexanecarboxyl‐CoA dehydrogenase from *Syntrophus aciditrophicus* (14.5 μmol min^−1^ mg^−1^) (Kung et al. 2013) or *Geobacter metallireducens* (29 μmol min^−1^ mg^−1^) (Kung et al. 2014). However, the low activity fits the comparatively slow growth of N47 with doubling times of 8 days (Heker, Haberhauer, and Meckenstock 2023).

### 4.3 Molecular Basis of the Substrate Specificity of ThnO for (1*R* ,2*R* )‐2‐Carboxycyclohexylacetyl‐CoA

ThnO exhibits stereospecific discrimination of one enantiomer of 2‐carboxycyclohexylacetyl‐CoA. To find out the configuration, the active substrate (in form of the non‐CoA derivative) was analysed by crystallography and vibrational circular dichroism (VCD) (Merten, Golub, and Kreienborg 2019), clearly demonstrating that ThnO specifically converts (1*R*,2*R*)‐2‐carboxycyclohexylacetyl‐CoA (compound 17 in Figures 4, 6 and S6).

The homology model of ThnO in complex with the substrate (1*R*,2*R*)‐2‐carboxycyclohexylacetyl‐CoA and FAD (Figure S12) indicated that the Cα‐Cβ bond of the substrate is sandwiched between the catalytic glutamate (363E) and the isoalloxazine ring of FAD, positioning it optimally for the α‐β dehydrogenation reaction. The carbonyl oxygen of the substrate is stabilised by the hydrogen bond with the amide nitrogen of the catalytic glutamate. The carboxy group of the dicarboxyl‐thioester is surrounded by polar or positively charged amino acids (N88 and R249). Such polar or positively charged residues have been observed in enzymes oxidising the dicarboxyl‐thioesters methylsuccinyl‐CoA and glutaryl‐CoA and participate in binding and stabilising the carboxy‐groups while orienting the substrate within the active site (Fu et al. 2004; Erb, Fuchs, and Alber 2009).

The binding cavity for the C3‐C4 atoms of the substrate (1*R*,2*R*)‐2‐carboxycyclohexylacetyl‐CoA in ThnO was sculpted by the proceeding amino acid (G362) of the catalytic glutamate (E363), which is usually a conserved Tyr involved in binding the substrates via main chain contact in other acyl‐CoA dehydrogenases. However, when accommodating an α‐substituted substrate, like isobutyryl‐CoA, benzylsuccinyl‐CoA, methylsuccinyl‐CoA, or cyclohexane‐1‐carbonyl‐CoA, this amino acid site changes to relatively smaller residues such as Phe, Leu, or Val (Figure S10B) (Leutwein and Heider 2002; Battaile et al. 2004; Erb, Fuchs, and Alber 2009; Kung et al. 2013). To accommodate the tertiary β‐carbon of substrates like iso(3)valeryl‐CoA or (1*R*,2*R*)‐2‐carboxycyclohexylacetyl‐CoA, iso(3)valeryl‐CoA dehydrogenase and ThnO respectively feature an even smaller Gly at the corresponding position (Figure S10B) (Tiffany et al. 1997). Therefore, the absence of the phenol ring of conserved Tyr in ThnO allows the bulky skeleton of 2‐carboxycyclohexylacetyl‐CoA to fit snugly in the wide cavity. The reduced activity of ThnT towards 2‐carboxycyclohexylacetyl‐CoA may result from the presence of a relatively larger Thr (T368) at this site.

### 4.4 The Intramolecular CoA‐Transferase ThnP

We demonstrated the activity of the CoA‐transferase ThnP, which performs an intramolecular CoA transfer between 2‐(carboxymethyl)cyclohexane‐1‐carboxyl‐CoA and 2‐carboxycyclohexylacetyl‐CoA. When the CoA thioester of 2‐(carboxymethyl)cyclohexane‐1‐carboxylic acid was prepared in cell free extract, it clearly exhibited a mixture of the two isomers (compounds 16 and 17 in Figure 4), indicating that the isomeric mixture is most likely also the intermediate in vivo. The CoA transferase ThnP now produces an equilibrium between the two isomers which is important for the following oxidation reaction by ThnO. Because the acyl‐CoA dehydrogenase ThnO can only convert the 2‐carboxycyclohexylacetyl‐CoA isomer, the CoA has to be steadily transferred from 2‐(carboxymethyl)cyclohexane‐1‐carboxyl‐CoA, which is the function of ThnP (Figures 7 and 8). We conclude that in the beta‐oxidation reactions after the first ring cleavage, an acetyl‐CoA moiety is cleaved off from the precursor 3‐(2‐[carboxymethyl]cyclohexyl)‐3‐oxopropionyl‐CoA (compound 7a in Figure 1B) which produces 2‐(carboxymethyl)cyclohexane‐1‐carboxyl‐CoA. This has to be converted to 2‐carboxycyclohexylacetyl‐CoA in the intramolecular CoA transfer reaction to allow for the subsequent beta‐oxidation of the carboxymethyl‐residue.

CoA transferases typically catalyse the intermolecular CoA transfer between disparate CoA donors and acceptors. In contrast, ThnP selectively catalyses an intramolecular CoA transfer but lacks the ability to utilise alternative CoA donors for intermolecular CoA transfer. The intramolecular transfer of the CoA moiety preserves the energy‐rich CoA thioester bond, preventing the release of the free acid or the transfer of CoA to other receptors, thereby avoiding the loss of intermediates and the need for additional ATP to activate the free acid (Pfister, Zarzycki, and Erb 2023), which makes intramolecular CoA transfer of 2‐(carboxymethyl)cyclohexane‐1‐carboxyl‐CoA in N47 emerge as an energetically highly efficient strategy.

The best‐studied intramolecular CoA transferases are succinyl‐CoA:phenylsuccinate CoA‐transferases (exhibits both inter and intramolecular transfer properties) involved in anaerobic metabolism of auxin in *A. aromaticum* and mesaconyl‐CoA C1‐C4 CoA transferases (specialised intramolecular transfer) from *C. aurantiacus* and *R. castenholzii* (Zarzycki et al. 2009; Schühle, Nies, and Heider 2016; Min et al. 2022). The above three enzymes belong to family III/Frc family CoA transferase (Figure 9), which raises the question of whether intramolecular CoA transfer characteristics evolved separately from a common ancestor.

The absence of the conserved active site glutamate in ThnP, which is typically found in family I CoA transferases, raises questions about its catalytic mechanism. According to the alpha fold model of ThnP, a glutamate E269 is in a topologically similar position with the active site E238 of 4‐hydroxybutyrate CoA‐transferase (Protein Data Bank entry 3GK7) belonging to the Cat1 family (Figure S13) (Macieira et al. 2012). However, the distance between E269 and the thiol sulfur of coenzyme A in ThnP is 6.5 Å, which is significantly larger than the corresponding distance between E238 and the thiol sulfur of coenzyme A in 4‐hydroxybutyrate CoA‐transferase. This shift in the position of the putative active site residue E269 in ThnP may help create a larger active cavity to accommodate the bulkier substrate 2‐(carboxymethyl)cyclohexane‐1‐carboxyl‐CoA. However, when comparing the position of active site aspartic acid in CoA transferases of the family III/Frc group, which also accommodate larger substrates, such as succinyl‐CoA: phthalate CoA transferase SptAB and succinyl‐CoA:phenylsuccinate CoA‐transferase IaaL, as well as Mct, which uses the smaller substrate mesaconyl‐CoA, no significant differences are observed in the position of their conserved active sites (Schühle, Nies, and Heider 2016; Mergelsberg, Egle, and Boll 2018; Pfister, Zarzycki, and Erb 2023). This suggests that the presumed active site E269 in ThnP is less likely to have shifted due to the substrate's larger structure.

An alternative explanation for the possibility that E269 facilitates the nucleophilic attack necessary for CoA transfer, ThnP may employ a novel mechanism that involves forming an internal anhydride intermediate, rather than an enzyme‐bound acyl‐glutamyl anhydride. The internal anhydride would be formed through nucleophilic attack by the acetate side chain of the substrate 2‐(carboxymethyl)cyclohexane‐1‐carboxy‐CoA on its acyl‐CoA carbonyl group, followed by the attack of CoA thiolate on the carbonyl of the internal anhydride adduct (Figure S14). This alternative mechanism can agree with the absence of the catalytic glutamate or aspartate residue within the appropriate range of nucleophilic attack.

A notable feature of the intermolecular CoA transferase formyl‐CoA: oxalate CoA‐transferase, belonging to family III/Frt group, is the presence of a flexible tetra‐glycine loop (Figure S11), ensuring that the receptor acid enters and exits the active site under the control of the flexible glycine ring (Berthold et al. 2008; Lee et al. 2010). However, this tetra‐glycine motif is absent in the intramolecular mesaconyl‐CoA transferase of family III/Frt group, which is thought to prevent conformational changes of the active site during reactions to exchange substrate and lead to a strong preference for intramolecular transfer (Min et al. 2022; Pfister, Zarzycki, and Erb 2023). Notably, this glycine loop is not conserved across other CoA transferases in the Family III/Frc group. For instance, it is absent in crotonobetainyl‐CoA:carnitine CoA‐transferase CaiB (intermolecular CoA transferase) in *E. coli*, succinyl‐CoA: phthalate CoA transferase SptAB (intermolecular CoA transferase) and succinyl‐CoA:phenylsuccinate CoA‐transferase IaaL (both inter‐ and intramolecular CoA transferase) in *A. aromaticum* (Figure S11) (Rangarajan et al. 2005; Schühle, Nies, and Heider 2016; Mergelsberg, Egle, and Boll 2018). This raises questions about the relationship between the presence of the glycine loop and the enzyme's selectivity for intermolecular versus intramolecular CoA transfer.

A similar flexible QXGhG loop (X for any amino acid and h for a hydrophobic amino acid) can be found in the intermolecular CoA transferases of the family I/Cat1 group (Figure S11) (Torres et al. 2011; Macieira et al. 2012; Mullins and Kappock 2012). For example, in 4‐hydroxybutyrate CoA‐transferase (4‐HB‐CoAT), the formation and disruption of hydrogen bonds between CoA and Ile216/Gln337 in 4‐HB‐CoAT during the reaction prompts a flip of the active site loop (from Gly215 to Ile219) (Macieira et al. 2012). This transition facilitates the toggling between closed and open conformations, which is crucial for enabling free acid transfer (Figure S13). However, the flexible QXGhG motif also exists in the corresponding position in ThnP (Figures S11 and S13), which theoretically not release free acceptor acid during the CoA transfer reaction. We speculate that the transformation of the flexible loop may create an extended pocket, allowing the bulky intermediate—whether a free dicarboxylic acid or an internal anhydride—to rotate within the pocket and passively redirect after CoA thiolate release. Since the structures of ThnP and mesaconyl‐CoA transferase differ significantly, and no intramolecular CoA transferases from family I have been studied so far, the functional importance of the flexible loop in ThnP remains unclear and requires further investigation with fast time scale protein motions analysis in the future.

### 4.5 Revised Pathway of Anaerobic Naphthalene Metabolism

Based on the identification of CoA‐transferase, acyl‐CoA dehydrogenase and its product 2‐carboxycyclohexylideneacetyl‐CoA, we hereby establish a modified metabolic pathway (Figure 1B) and conclude that the upstream naphthalene degradation pathway produces the 2‐(carboxymethyl)cyclohexane‐1‐carboxyl‐CoA isomer, which has consequences for the interpretation of earlier data concerning the cleavage of ring I of naphthoic acid (Weyrauch et al. 2020). The central intermediate naphthoyl‐CoA is first reduced by two type III aryl‐CoA reductases to tetrahydro‐naphthoyl‐CoA followed by a type I aryl‐CoA reductase (tetrahydro‐naphthoyl‐CoA reductase) where the remaining benzene ring is reduced to a diene. The diene can theoretically appear as two isomers, the 4,4a,5,6,7,8‐hexahydro‐2‐naphthoyl‐CoA (compound 2a in Figure 1B) and the 4a,5,6,7,8,8a‐hexahydro‐2‐naphthoyl‐CoA isomer (compound 2b), which would lead to different reactions and produce a same intermediate 1,3‐dioxodecahydro‐2‐naphthoyl‐CoA (compound 6). The ring I of 1,3‐dioxodecahydro‐2‐naphthoyl‐CoA then cleavages between C1C2 or C2C3 producing 3‐(2‐[carboxymethyl]cyclohexyl)‐3‐oxopropionyl‐CoA (compound 7a) or 4‐(2‐carboxycyclohexyl)‐3‐oxobutyryl‐CoA (compound 7b), respectively. Consequently, a thiolysis reaction shortens one of the side chains by an acetyl unit producing 2‐(carboxymethyl)cyclohexane‐1‐carboxyl‐CoA (compound 8a) or 2‐(2‐carboxycyclohexyl)‐acetyl‐CoA (compound 8b). Because we showed here that the pathway proceeds via 2‐(carboxymethyl)cyclohexane‐1‐carboxyl‐CoA, we accordingly propose the pathway depicted in Figure 1 and conclude that the cleavage of ring I of naphthoic acid is achieved by breaking the bond between C2 and C3 of the intermediate 1,3‐dioxodecahydro‐2‐naphthoyl‐CoA (compound 6) to produce 3‐(2‐[carboxymethyl]cyclohexyl)‐3‐oxopropionyl‐CoA (compound 7a). The product of tetrahydro‐naphthoyl‐CoA reductase and the pathway producing 1,3‐dioxodecahydro‐2‐naphthoyl‐CoA still remain unclear.

Here, we demonstrated that the downstream pathway of anaerobic naphthalene degradation started with 2‐(carboxymethyl)cyclohexane‐1‐carboxyl‐CoA where the intramolecular CoA‐transferase ThnP produces 2‐carboxycyclohexylacetyl‐CoA. The following dehydrogenation is catalysed by ThnO at the acetyl‐CoA thioester. This reaction is probably followed by a hydratase and a novel lyase reaction acting at an intermediate with a tertiary hydroxyl‐group to open the ring. The elucidation of the reactions catalysed by ThnO and ThnP sheds further light on the anaerobic naphthalene degradation pathway at the point initiating the second ring cleavage.

## Author Contributions

**Yachao Kong:** methodology, investigation, validation, formal analysis, writing – review and editing, writing – original draft. **Jan Riebe:** investigation, methodology, formal analysis, writing – original draft, writing – review and editing. **Malte Feßner:** investigation, methodology, formal analysis, writing – original draft. **Torsten Schaller:** methodology, investigation, formal analysis, writing – original draft. **Christoph Wölper:** investigation, formal analysis, writing – original draft. **Florian Stappert:** investigation, formal analysis, writing – original draft. **Sven W. Meckelmann:** methodology, writing – original draft. **Matthias Krajnc:** investigation. **Philip Weyrauch:** investigation, writing – review and editing. **Oliver J. Schmitz:** conceptualization, supervision, writing – review and editing. **Christian Merten:** conceptualization, supervision, writing – review and editing. **Jochen Niemeyer:** conceptualization, supervision, writing – review and editing. **Xiaoke Hu:** funding acquisition, supervision, writing – review and editing. **Rainer U. Meckenstock:** conceptualization, funding acquisition, supervision, project administration, writing – review and editing.

## Conflicts of Interest

The authors declare no conflicts of interest.

## Supporting information


Data S1.

## Data Availability

The data supporting the findings of this study can be found in the Appendix. The crystallographic data for this paper are openly available in The Cambridge Crystallographic Data Centre (accession number CCDC‐2369957): https://www.ccdc.cam.ac.uk/structures/Search?Ccdcid=2369957.
