# Supplementary material for: A CoA‐Transferase and Acyl‐CoA Dehydrogenase Convert 2‐(Carboxymethyl)cyclohexane‐1‐Carboxyl‐CoA During Anaerobic Naphthalene Degradation
Source: Environ Microbiol. 2024 Dec 19;26(12):e70013. doi: 10.1111/1462-2920.70013 (PMC11659651; doi:10.1111/1462-2920.70013)
Supplement: Supplementary file 1 — Data S1. [file EMI-26-e70013-s001.docx]

APPENDIX

**
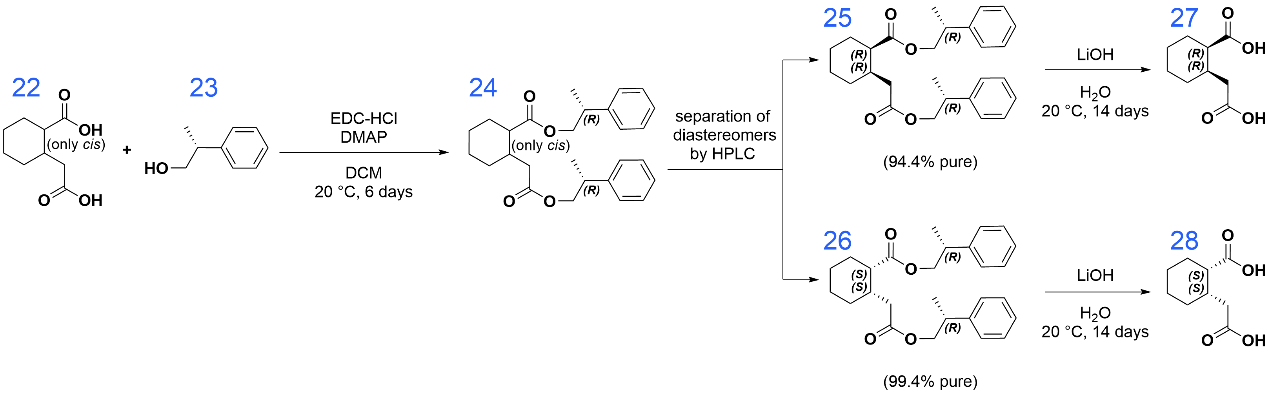
I. Appendix Figures:**

**Figure S1.** Reaction scheme for derivatization of racemic acid *cis*-2-(carboxymethyl)cyclohexane-1-carboxylic acid (22) with chiral alcohol (*R*)-2-phenyl-1-propanol (23) to a pair of diastereomeric diesters with (*S*,*S*,*R*,*R*) and (*R*,*R*,*R*,*R*) configuration (1:1 mixture) (24), which could be separated by chiral HPLC and were then hydrolysed to give samples of pure enantiomers of *cis*-2-(carboxymethyl)cyclohexane-1-carboxylic acid (27 and 28).


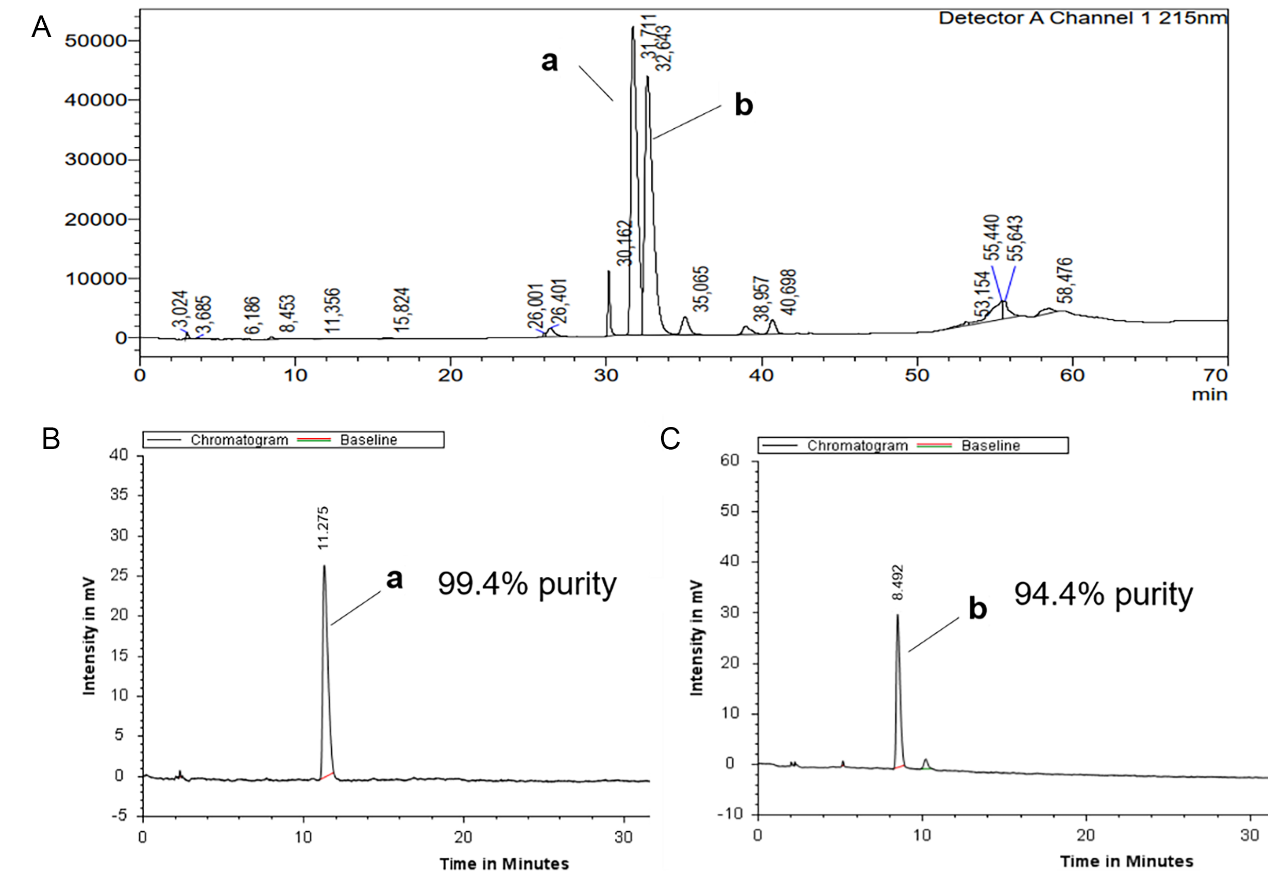
**Figure S2.** A) HPLC-chromatogram for preparative-scale separation of the diastereomers of 2-phenylpropyl-2-(2-oxo-2-(-2-phenylpropoxy)ethyl)cyclohexane-1-carboxylate (diastereomer-a elutes at 31.7 minutes and diastereomer-b elutes at 32.6 minutes) and chromatograms of analytical HPLC of the collected fractions: B) diastereomer-a elutes at 11.3 minutes; C) diastereomer-b elutes at 8.5 minutes (both preparative and analytical scale separations used Daicel Chiralpak IA stationary phases and *n*-hexane/2-propanol as eluent). Note that the order of elution between preparative and analytical HPLC is reversed.


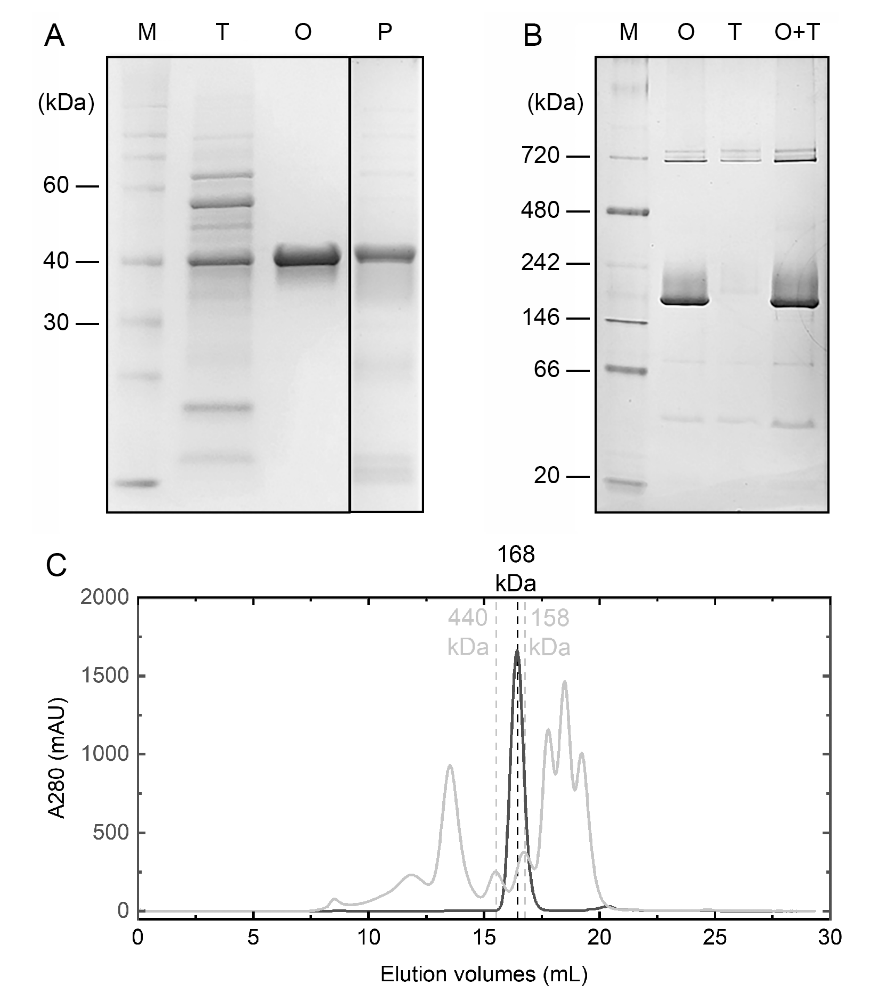
**Figure S3.** Heterologous production and purification of recombinant acyl-CoA dehydrogenase ThnO, ThnT and CoA transferase ThnP, and determination of complex size of ThnO. A) 12% SDS-PAGE of purified acyl-CoA dehydrogenases ThnO/ThnT and CoA transferase ThnP. Lanes: T, 6.9 μg of protein ThnT; O, 4.4 μg of protein ThnO; P, 4.2 μg of protein ThnP. B) 4-16% Bis-tris Blue Native-PAGE of individual ThnO or ThnT, or mixture of ThnO and ThnT. Lanes: O, 12 μg of protein ThnO; T, 2 μg of protein ThnT; O+T, 12 μg of protein ThnO and 2 μg of protein ThnT. Number at the Y-achses indicate the molecular masses of standard proteins in lane M. C) Size exclusion chromatography of purified ThnO complex. The chromatogram of ThnO is shown as a black line, while calibrations with protein molecular mass standards are shown in grey. The two grey dashed lines correspond to standards with molecular masses of 158 kDa and 440 kDa, respectively. The molecular mass of ThnO was determined to be 168 kDa after comparison with the calibration curve. All the purified proteins mentioned above are from the elution step of protein purification.

**
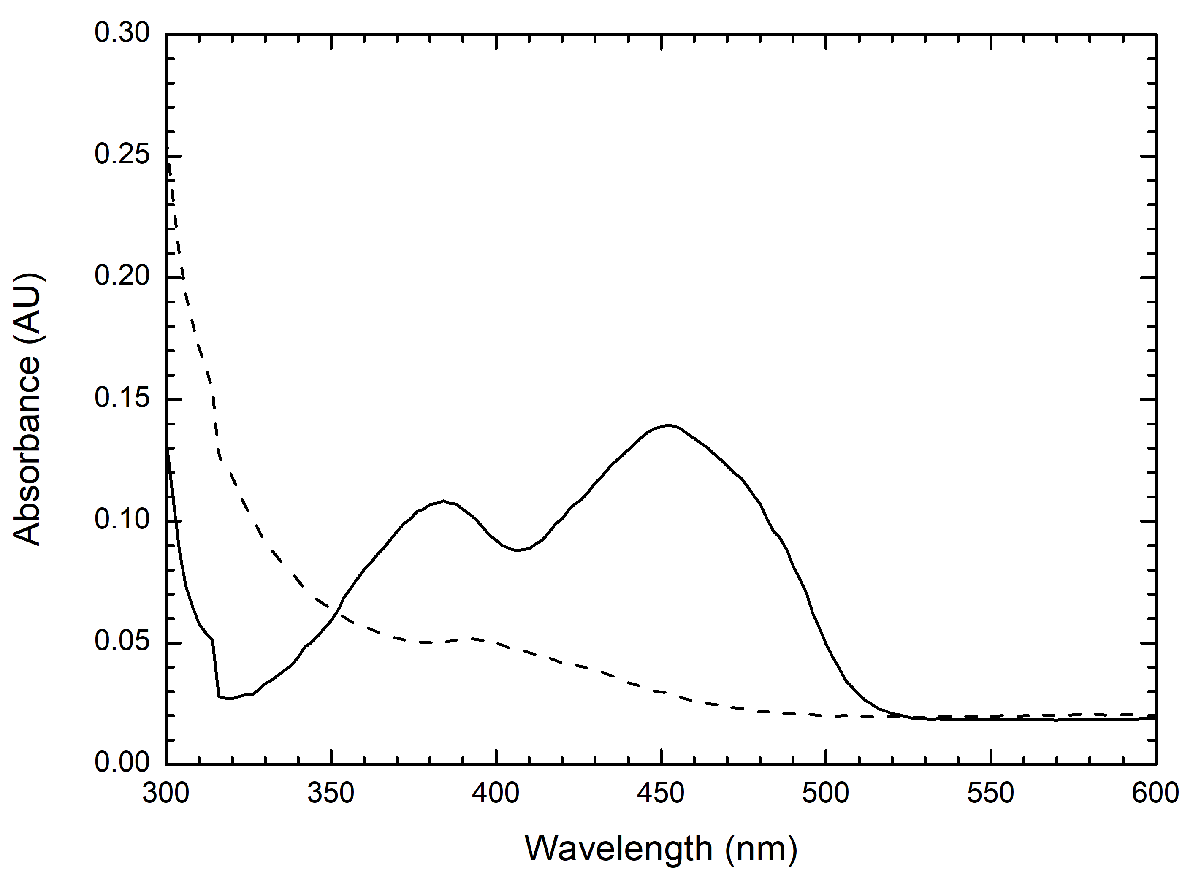
Figure S4.** UV-vis spectra of heterologously expressed and purified ThnO before (solid lines) and after (dashed lines) reduction with sodium dithionite. Absorbance maxima of the oxidized enzyme are at 380 nm and 450 nm.

**
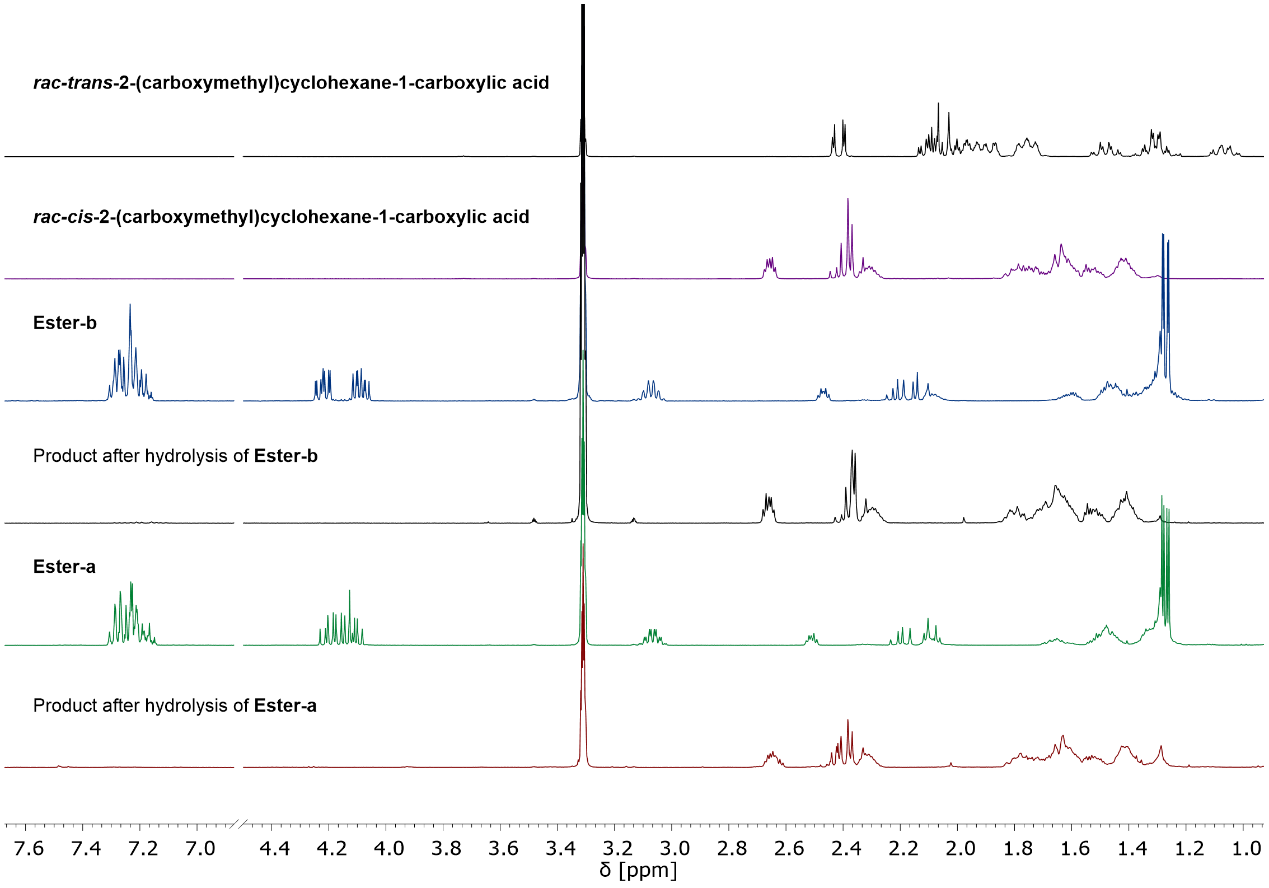
**

**Figure S5.** Stacked ^1^H-NMR spectra of racemic-2-(carboxymethyl)cyclohexane-1-carboxylic acid, the purified diastereomeric esters a and b and the resulting enantiopure acids after hydrolysis (all 400 MHz, MeOD, 298 K). In absence of additional chiral information, the spectra of the racemate and the enantiopure acids are all identical.


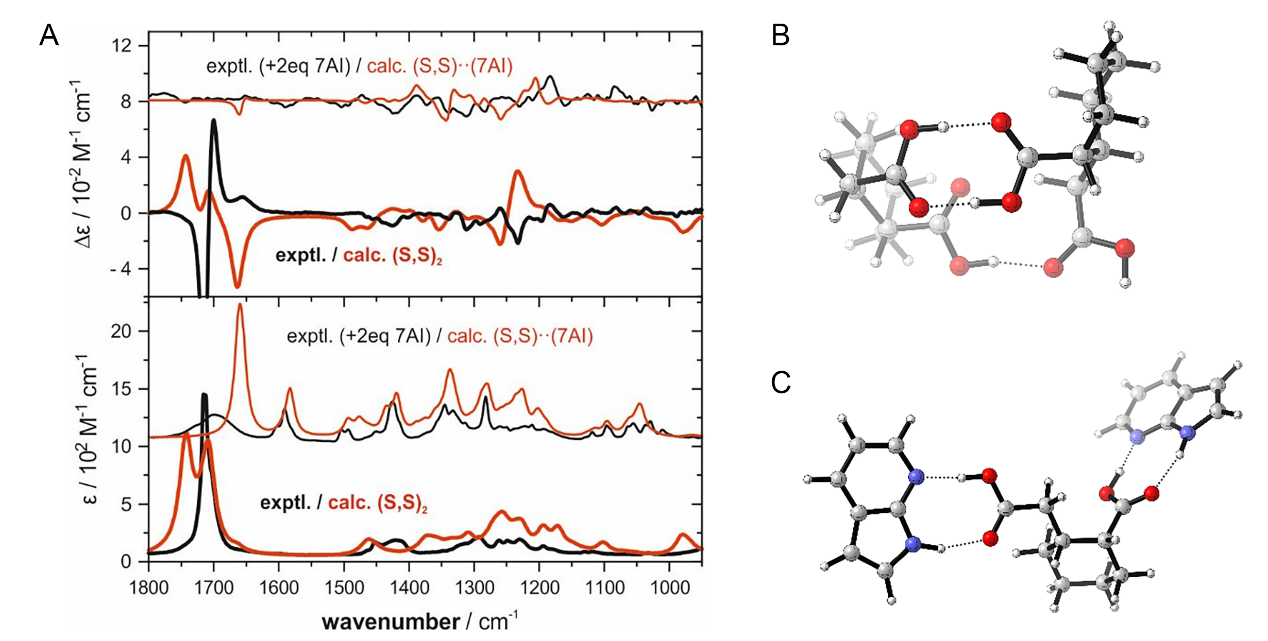


**Figure S6.** Vibrational circular dichroism (VCD) and infrared spectroscopy (IR) spectra of the enantiopure acid of the real substrate of dehydrogenase ThnO ((1*R*,2*R*)-2-(carboxymethyl)cyclohexane-1-carboxylic acid). Left: Comparison of the experimental (in black) and computed (in red) VCD (upper panel) and IR spectra (lower panel). Note that the computed spectra are presented for the mirror image structure 7AI-complexes with (1*S*,2*S*)-2-(carboxymethyl)cyclohexane-1-carboxylic acid to emphasize the mirror-image relation. Right: Lowest energy conformations of the dimeric structure of (1*R*,2*R*)-2-(carboxymethyl)cyclohexane-1-carboxylic acid (B) and 7(AI)_2_-complex with (1*R*,2*R*)-2-(carboxymethyl)cyclohexane-1-carboxylic acid (C). 7AI: 7-azaindole.

**
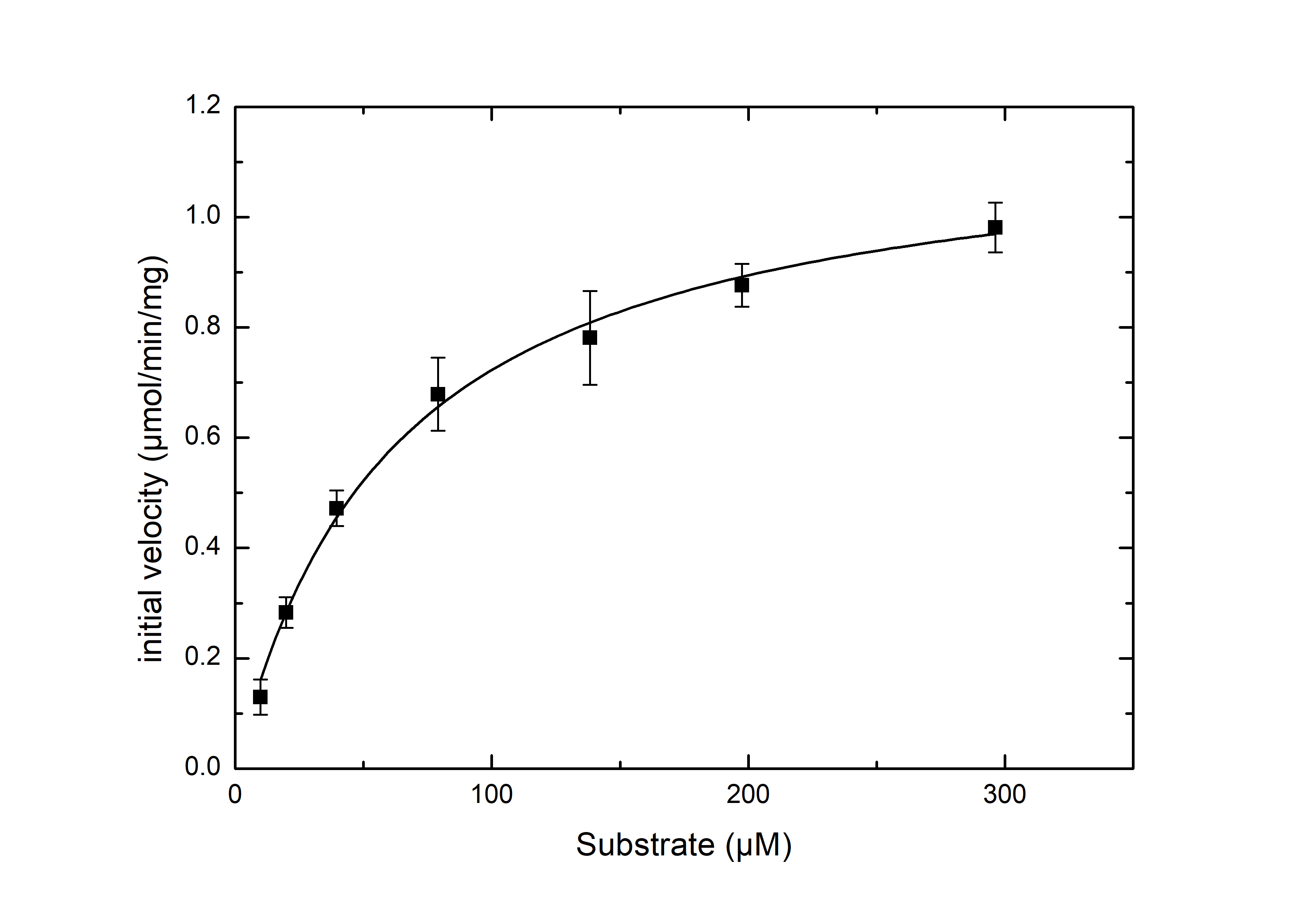
**

**Figure S7.** Michaelis–Menten plot of acyl-CoA dehydrogenase ThnO with various concentrations of substrate cis-2-(carboxymethyl)cyclohexane-1-carboxylic acid CoA thioester.


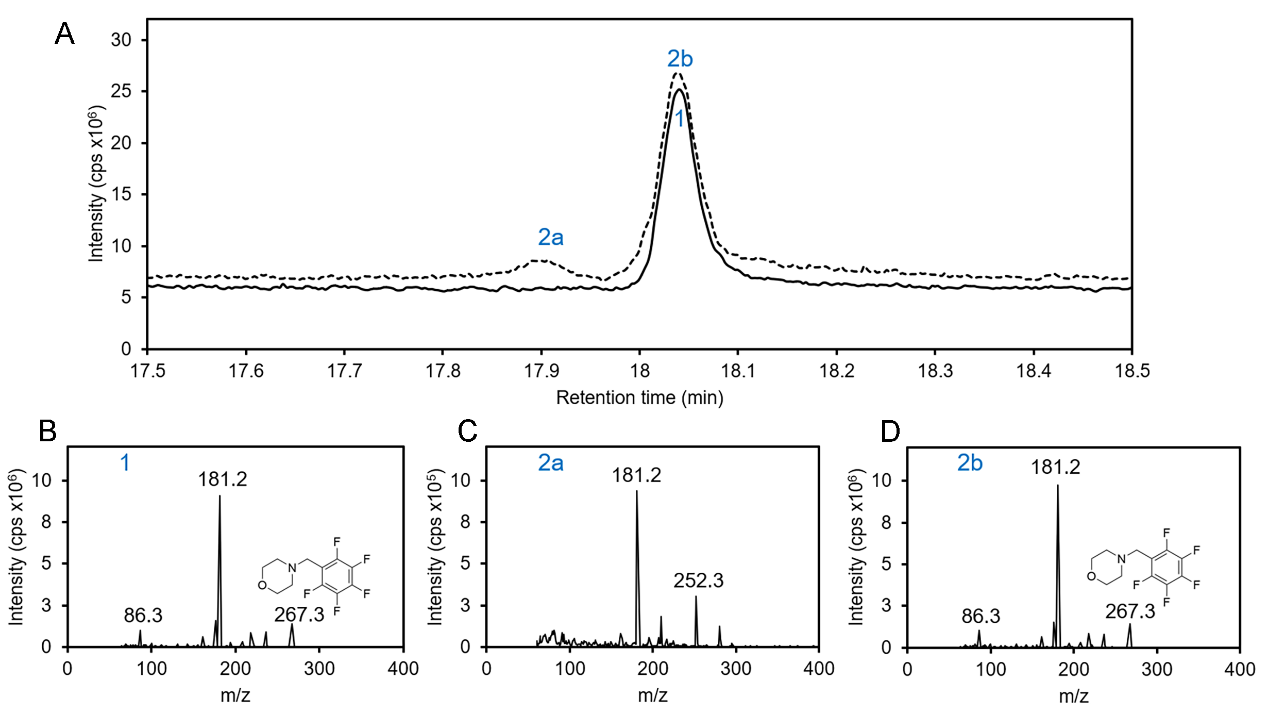


**Figure S8.** GC-MS chromatograms and spectra of pentafluorobenzyl bromide (PFB-Br) derivatives of 2-(carboxymethyl)cyclohexane-1-carboxylic acid CoA ester derived from N47 cell-free extracts before and after treatment with heterologously expressed dehydrogenase ThnO.

A) GC-MS chromatograms of PFB derivatives eluted at 18 min. Dotted line: before treatment with ThnO; Solid line: after treatment with ThnO. B) C) and D) GC-MS spectra of PFB derivatives eluted at 18 min of peak 1, 2a and 2b in panel A. Both of the spectra of peak 1 and 2b are identified as PFB-derived species N-(2,3,4,5,6-Pentafluorobenzyl)morpholine. No PFB-derivatized 2-(carboxymethyl)cyclohexane-1-carboxyl-CoA or 2-(2-carboxycyclohexyl)acetyl-CoA can be found and there are no qualitative differences in the fragment spectrum before and after treatment with ThnO.


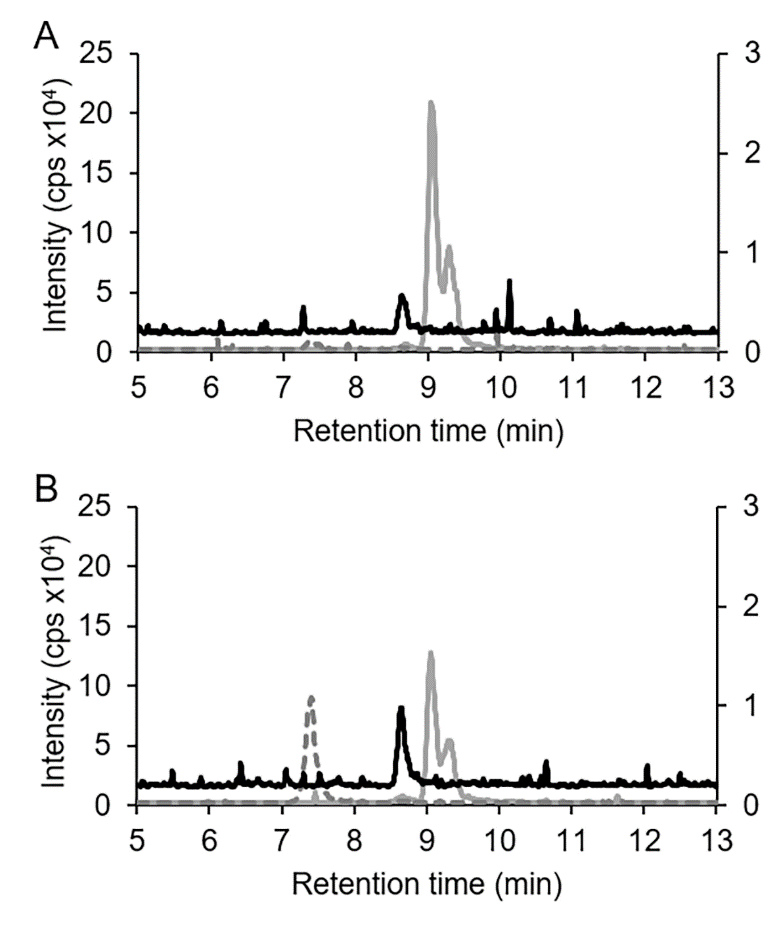
**Figure S9.** LC-MS chromatograms showing the conversion of *cis*-2-(carboxymethyl)cyclohexane-1-carboxylic acid CoA esters (four isomers) in cell free extract of culture N47. A) conversion of the substrate at t=0 min. B) t=5 min. Grey lines: left y-axes, ion counts for *cis*-2-(carboxymethyl)cyclohexane-1-carboxylic acid CoA esters with m/z = 936. Black lines: right y-axes, ion counts of conversion product with m/z = 934. Dot lines: left y-axes, ion counts of conversion product 2-(1-hydroxy-2-carboxycyclohexyl)acetyl-CoA with m/z = 952.

**
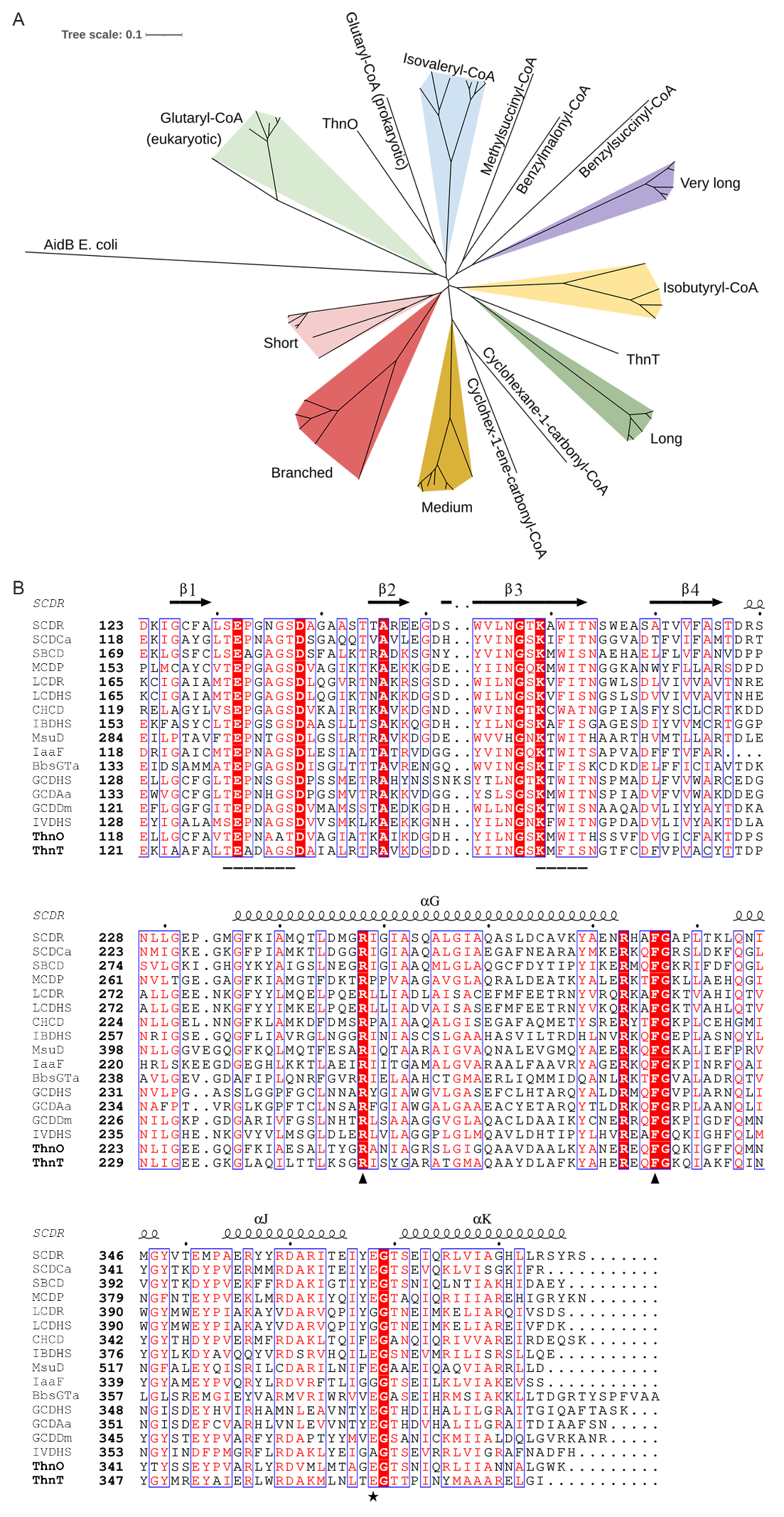
**

**Figure S10.** A) Neighbour-joining tree of flavin-dependent acyl-CoA dehydrogenase homologs composed of biochemically characterized flavin-dependent acyl-CoA dehydrogenases from the Swiss-Prot database and ThnO/ThnT (shown in bold). AidB was chosen as an outgroup. B) Multiple sequence alignment of ThnO/ThnT with various flavin-dependent acyl-CoA dehydrogenases. SCDR and SCDCa, SBCD, MCDP, LCDR and LCDHS, acyl-CoA dehydrogenase from rat and *Clostridium acetobutylicum* (short-chain, accession number 1JQI_A and P52042), rat (short-branched, P70584), pig (medium-chain, NP_999204), rat and human (long-chain, P15650 and P28330), respectively; CHCD, cyclohexane-1-carbonyl-CoA from *Syntrophus aciditrophicus* (Q2LQP0); IBDHS, human isobutyryl-CoA dehydrogenase (NP_055199); MsuD, (2S)-methylsuccinyl-CoA dehydrogenase from *Rhodobacter sphaeroides* (YP_351728.2); IaaF, benzylmalonyl-CoA dehydrogenase from *Aromatoleum aromaticum* (WP_011236983); BbsGTa, (R)-benzylsuccinyl-CoA dehydrogenase from *Thauera aromatica* (AAF89842); GCDHS, GCDAa and GCDDm, glutaryl-CoA dehydrogenase from human (1SIR_A), *A. aromaticum* (CAI07810) and *Desulfococcus multivorans* (C3UVB0); IVDHS, human isovaleryl-CoA Dehydrogenase (1IVH_A). α-Helices are named alphabetically, β-sheets are numbered. Catalytic glutamates are marked with a star (except isovaleryl-CoA and long-chain acyl-CoA dehydrogenase). Residues that are reported to be involved in the binding of FAD are underlined. The residues for positioning the CoA moiety are highlighted by a triangle.

**
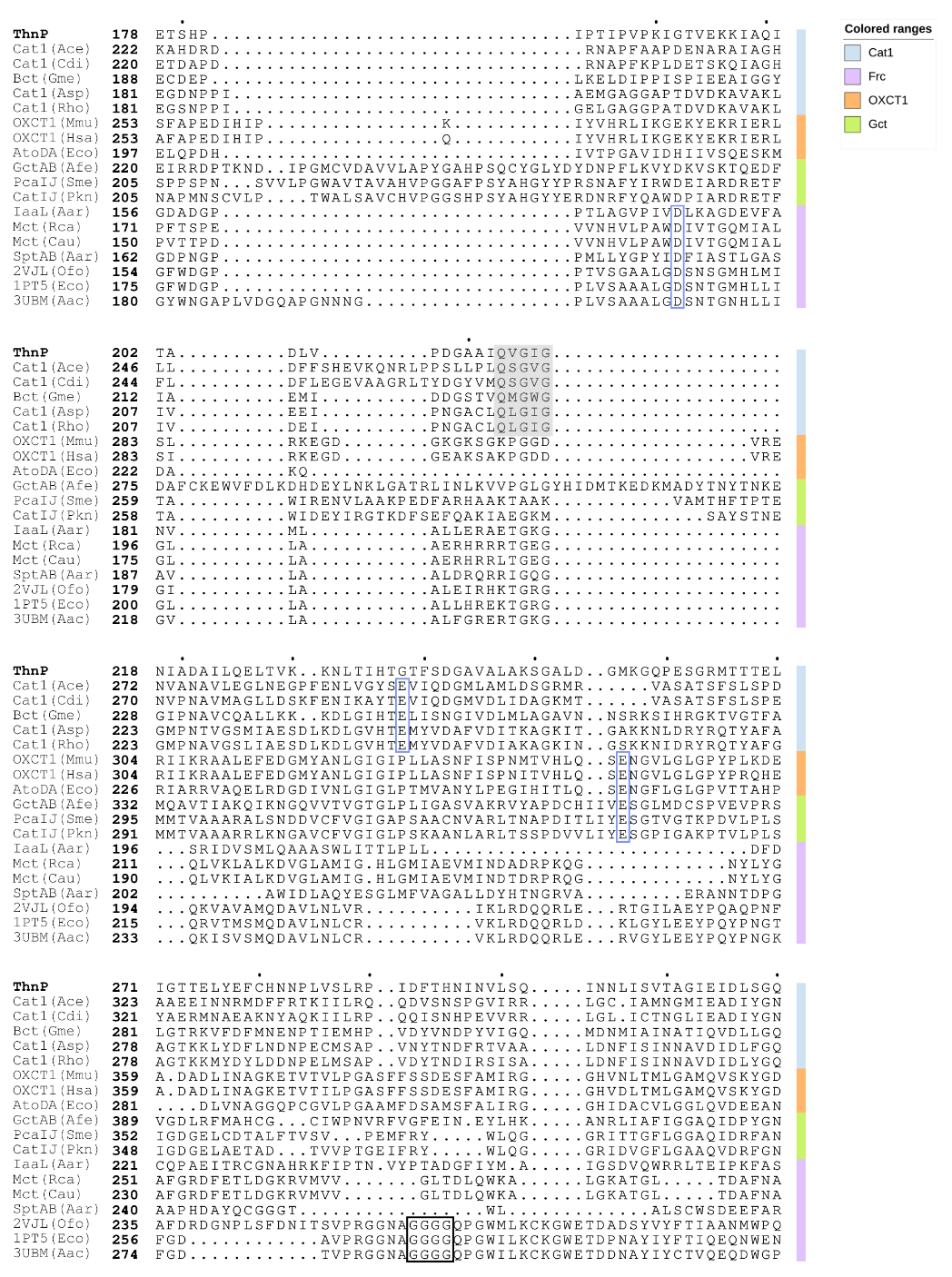
Figure S11.** Multiple sequence alignment of CoA-transferase ThnP (shown in bold) and members of various CoA transferase families. The subfamily of each sequence is marked with a different colour after the sequence. The active sites of CoA transferases are highlighted with blue boxes. The conserved flexible QXGhG motif in the Cat1 family including ThnP is marked with a grey shadow. The flexible tetra-glycine loop in formyl-CoA:oxalate CoA-transferase from *Oxalobacter formigenes, Escherichia coli,* and *Acetobacter aceti* (named as 2VJL, 1PT5, and 3UBM in this figure) are highlighted with a black box. The mesaconyl-CoA transferases lacking this tetra-glycine motif at the corresponding position are named Mct(Rca) and Mct(Cau) in this figure.


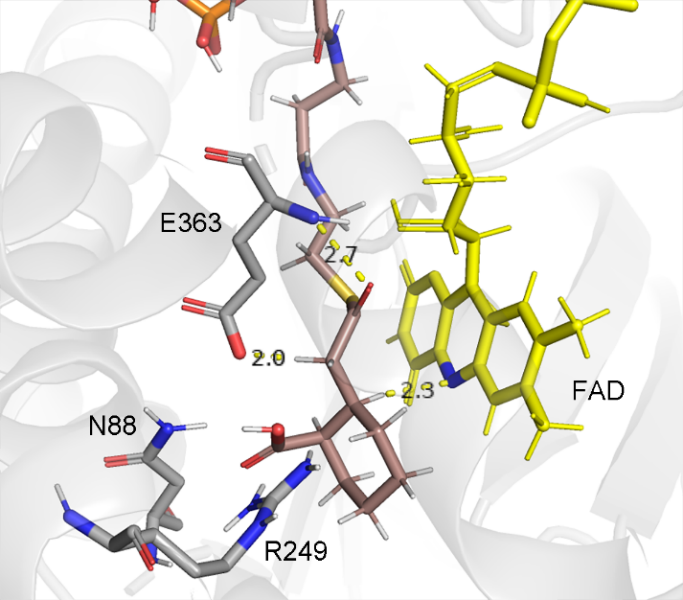
**Figure S12.** Homology model of the active center of (1R, 2R)-2-carboxycyclohexylacetyl-CoA dehydrogenase (ThnO) in complex with (1R, 2R)-2-carboxycyclohexylacetyl-CoA and FAD. The structure of the substrate is drawn in brown, and the oxygen atoms of the carboxylate residue and carbonyl group are drawn in red. The structure of FAD is shown in yellow and the N5 atom of the isoalloxazine ring of FAD which accepts the hydride transferred during the oxidation reaction is depicted in blue. The catalytic glutamic acid of ThnO (E363) and the amino acids for stabilizing the carboxyl group (N88 and R249) are shown in silver, the oxygen and nitrogen atoms are drawn in red and blue, respectively. Distances to selected amino acids in the surroundings are given.


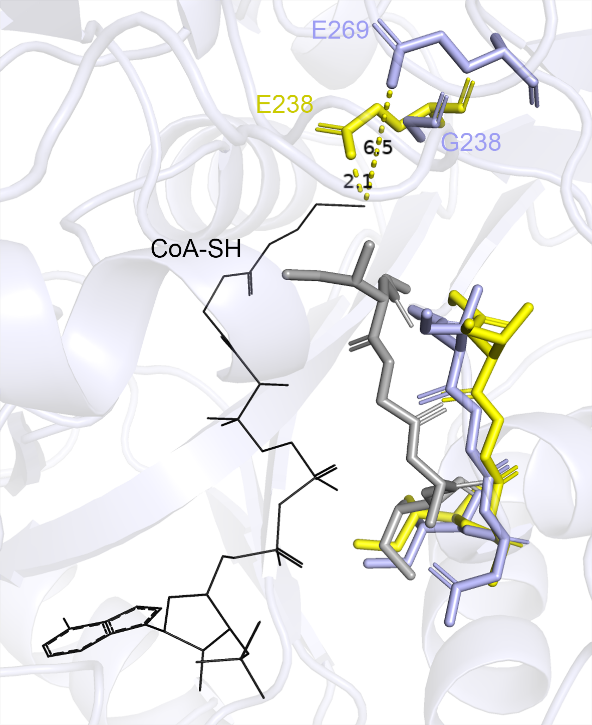
**Figure S13.** Homology model of the active center of CoA transferase ThnP and comparison with the crystal structure of 4-hydroxybutyrate CoA-transferase (4-HB-CoAT) from *Clostridium aminobutyricum* in the open (Protein Data Bank accession number 3GK7) and closed conformation (Protein Data Bank accession number 3QDQ) in complex with CoA-SH. The carbon skeleton of residues from ThnP, and the open or closed conformation of 4-HB-CoAT are drawn in light blue, yellow and grey, respectively. The structure of CoA-SH is drawn in black. The distances from the residue E238 of 4-HB-CoAT and E269 of ThnP to the thiol sulfur of coenzyme A are shown.

**
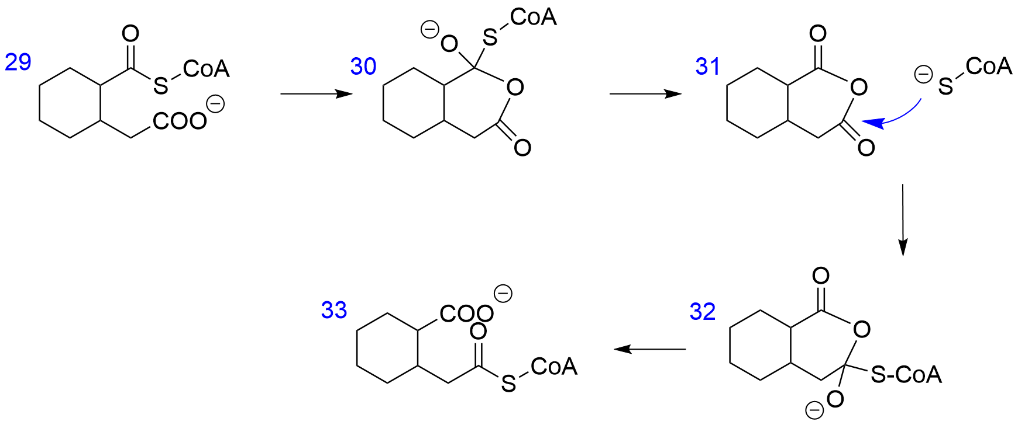
Figure S14.** The proposed reaction mechanism of ThnP is initiated by a nucleophilic attack by the acetate side chain of the substrate 2-(carboxymethyl)cyclohexane-1-carboxyl-CoA (29) on its acyl-CoA carbonyl, instead of the nucleophilic attack by the key glutamate side chain conserved in the Cat1 family CoA transferase. This is followed by an attack of CoA thiolate on the carbonyl of the internal anhydride adduct (31), ultimately resulting in the formation of the product 2-carboxycyclohexylacetyl-CoA (33).

**Ⅱ. Appendix Tables:**

**Table S1.** List of *thn* genes in N47 genome and the hypothetical functions of the encoded enzymes

| Gene | | ORF in N47 | Hypothetical function | Naming in a previous work^17^ |
| --- | --- | --- | --- | --- |
| *thnR* | N47_E41510 | | transcriptional regulator | ORF27 |
| *thnA* | N47_E41500 | | enoyl-CoA hydratase/isomerase | ORF28 |
| *thnB* | N47_E41490 | | 5,6,7,8-tetrahydro-2-naphthoyl-CoA reductase | *ncrC* |
| *thnC* | N47_E41480 | | 5,6,7,8-tetrahydro-2-naphthoyl-CoA reductase | *ncrB* |
| *thnD* | N47_E41470 | | 5,6,7,8-tetrahydro-2-naphthoyl-CoA reductase | *ncrA* |
| *thnE* | N47_E41460 | | 5,6,7,8-tetrahydro-2-naphthoyl-CoA reductase | *ncrD* |
| *thnF* | N47_E41450 | | ferredoxin | ORF33 |
| *thnG* | N47_E41430 | | oxidoreductase | ORF34 |
| *thnH* | N47_E41420 | | MaoC family dehydratase | ORF35 |
| *thnI* | N47_E41410 | | MaoC family dehydratase | ORF36 |
| *thnJ* | N47_E41400 | | thiolase | ORF37 |
| *thnK* | N47_E41390 | | β-hydroxyacyl-CoA dehydrogenase | ORF38 |
| *thnL* | N47_E41380 | | enoyl-CoA hydratase/isomerase | ORF39 |
| *thnM* | N47_E41370 | | enoyl-CoA hydratase/isomerase | ORF40 |
| *thnN* | N47_E41360 | | metallo-dependant hydrolase | ORF41 |
| *thnO* | N47_E41350 | | acyl-CoA dehydrogenase | ORF42 |
| *thnP* | N47_E41340 | | CoA-transferase | ORF43 |
| *thnQ* | N47_E41330 | | AtuA-like lyase | ORF44 |
| *thnS* | N47_E41320 | | AtuA-like lyase | ORF45 |
| *thnT* | N47_E41310 | | acyl-CoA dehydrogenase | ORF46 |
| *thnU* | N47_E41300 | | enoyl-CoA hydratase/isomerase | ORF47 |
| *thnV* | N47_E41290 | | thiolase | ORF48 |

**Table S2.** List of primers used

| Gene | Tag | Primer | Sequence |
| --- | --- | --- | --- |
| *thnO* | His6 | forward | ATATCCATGGATTTTCAATTAGAGCCTGAACTTG |
|  |  | reverse | ATATCTCGAGCTTCCAACCTAACGCGTTGTTTG |
|  | Twin-strep | forward | AGCGCGTCTCCAATGGATTTTCAATTAGAGCCTGAACTTG |
|  |  | reverse | AGCGCGTCTCCTCCCCTTCCAACCTAACGCGTTG |
| *thnT* | His6 | forward | ATATCCATGGATTTTGAGCTTTCTGAAGAACAG |
|  |  | reverse | ATATCTCGAGTATACCAAGTTCTCTTGCCGCAG |
|  | Twin-strep | forward | AGCGCGTCTCCAATGGATTTTGAGCTTTCTGAAGAACAG |
|  |  | reverse | AGCGCGTCTCCTCCCTATACCAAGTTCTCTTGCCG |
| *thnP* | His6 | forward | ATATTCATGAAATATGGAACTAAACCAAAAACAGG |
|  |  | reverse | ATATCTCGAGACTGTTTATCTTATTGAAAGCACTTTTTAG |

**Table S3.** Crystal data and structure refinement for the enantiopure non-CoA derivative of the real substrate of dehydrogenase ThnO ((1*R*,2*R*)-2-(carboxymethyl)cyclohexane-1-carboxylic acid)

| Empirical formula | C_9_H_14_O_4_ |
| --- | --- |
| CCDC Deposition Number | 2369957 |
| Formula weight | 186.20 |
| Density (calculated) | 1.333 g⋅cm^-3^ |
| *F*(000) | 800 |
| Temperature | 100(2) K |
| Crystal size | 0.195 × 0.146 × 0.132 mm^3^ |
| Crystal colour | pale yellow |
| Crystal description | tablet |
| Wavelength | 1.54178 Å |
| Crystal system | orthorhombic |
| Space group | *P*2_1_2_1_2_1_ |
| Unit cell dimensions |  |
| *a* [Å] | 8.4050(5) |
| *b* [Å] | 12.1600(7) |
| *c* [Å] | 18.1580(10) |
| *α* [°] | 90 |
| *β* [°] | 90 |
| *γ* [°] | 90 |
| Volume | 1855.83(18) Å^3^ |
| *Z* | 8 |
| Cell measurement reflections used | 9982 |
| Cell measurement *θ* min/max | 5.80°/80.02° |
| Diffractometer control software | Bruker APEX3(v2017.3-0) |
| Diffractometer measurement device | Bruker D8 Venture (Photon II detector) |
| Diffractometer measurement method | Data collection strategy APEX 3/Queen |
| *θ* range for data collection | 4.376°- 80.481° |
| Completeness to *θ* = 67.679° | 100.0% |
| Completeness to *θ*_max_ = 80.481° | 100.0% |
| Index ranges | -10 ≤ *h* ≤ 10 |
|  | -15 ≤ *k* ≤ 15 |
|  | -23 ≤ *l* ≤ 23 |
| Computing data reduction | Bruker APEX3(v2017.3-0) |
| Absorption coefficient | 0.877 mm^-1^ |
| Absorption correction | Semi-empirical from equivalents |
| Computation absorption correction | SADABS |
| Max./min. Transmission | 0.75/0.62 |
| *R*_merg_ before/after correction | 0.1096/0.0734 |
| Computing structure solution | Bruker APEX3(v2017.3-0) |
| Computing structure refinement | SHELXL-2017/1 |
| Refinement method | Full-matrix least-squares on *F*^2^ |
| Reflections collected | 98393 |
| Independent reflections | 4061 |
| *R*_int_ | 0.0481 |
| Reflections with *I* > 2*σ*(*I*) | 3953 |
| Restraints | 2 |
| Parameter | 260 |
| GooF | 1.052 |
| Weighting details | *w* = 1/[*σ*^2^(*F*_obs_^2^) + (0.0352P)^2^ + 0.4245P] |
|  | where P = (*F*_obs_^2^ + 2*F*_calc_^2^)/3 |
| *R*_1_ [*I* > 2*σ*(*I*)] | 0.0278 |
| *wR*_2_ [*I* > 2*σ*(*I*)] | 0.0721 |
| *R*_1_ [all data] | 0.0288 |
| *wR*_2_ [all data] | 0.0732 |
| Absolute structure parameter | 0.04(3) |
| Largest diff. peak and hole | 0.201/-0.129 |

**III. Detailed methods**

**Synthesis of potential metabolites and elucidation of enantiomeric structure**

*Racemic cis-isomer* (1*R*,2*R*/1*S*,2*S*) *and trans-isomer* (1*R*,2*S*/1*S*,2*R*) *of 2-(carboxymethyl)cyclohexane-1-carboxylic acid were synthesized earlier as described before* (Weyrauch et al., 2017).

*Derivatization and resolution of enantiomers of cis-2-(carboxymethyl)cyclohexane-1-carboxylic acid. Rac*-*cis*-2-(carboxymethyl)cyclohexane-1-carboxylic acid (60.0 mg, 322 µmol, 1 eq.), *N*,*N*-dimethylaminopyridine (7.9 mg, 64.5 µmol, 0.2 eq.), *N*-(3-dimethylaminopropyl)-*N*′-ethylcarbodiimide hydrochloride (135.9 mg, 709 µmol, 2.2 eq.) and (*R*)-2-phenyl-1-propanol (122.9 mg, 902 µmol, 2.8 eq.) were dissolved in dichloromethane (1.5 mL) and stirred for 24 hours at room temperature (Fig. S1). The mixture was diluted with dichloromethane (10 mL) and washed with 1M aqueous hydrochloric acid, saturated aqueous sodium bicarbonate and water (each 1 x 10 mL). The organic phase was separated, dried over sodium sulfate, filtered and solvent was removed under reduced pressure. The desired di-ester 2-phenylpropyl-2-(2-oxo-2-(-2-phenylpropoxy)ethyl)cyclohexane-1-carboxylate (compounds 25 and 26 in Fig. S1) was obtained as a mixture of diastereomers as a colourless oil (130 mg, 308 µmol, 95.4% yield, 1:1 ratio of diastereomers). The diastereomers were separated by preparative chiral stationary phase HPLC (a SCL-40 controller, a SPD-40V UV-VIS detector, and a LC-20AP high-pressure pump, Shimadzu) on a Daicel IA column (20 x 250 mm, 5 µm particle size) with an eluent of *n*-hexane/2-propanol (99/1), detection wavelength 215 nm and a flowrate of 20 mL/min. Purity of collected fractions was tested by analytical chiral stationary phase HPLC with a Smartline UV Detector 2600 (Knauer, Berlin, Germany) on a Daicel IA-3 column (4.6 x 250 mm, 3 µm particle size) with an eluent of *n*-hexane/2-propanol (99/1) at a flowrate of 1.5 mL/min. Fractions with >95% purity were combined.

Diastereomerically pure di-ester a or b (Fig. S2) (47.3 mg) were dissolved in methanol-*d*_4_ (1 mL) in an NMR-tube and a 1M aqueous solution of lithium hydroxide (0.5 mL) was added. The mixture was left at room temperature with occasional shaking until full conversion of the di-ester was observed by ^1^H-NMR spectroscopy (14 days). The reaction mixture was diluted with 2 M aqueous sodium hydroxide (5 mL) and extracted with dichloromethane (2 x 5 mL) to remove the formed alcohol. The aqueous phase was then acidified with 2 M hydrochloric acid and extracted with methyl-*tert*-butyl ether (3 x 5 mL). The combined ether extracts were dried over sodium sulfate, filtered and evaporated *in vacuo* to yield the enantiopure di-acid as a colourless oil (15.2 mg, 81.6 µmol, 72.9% isolated yield for di-ester a; 14.7 mg, 78.9 µmol, 70.5% isolated yield for di-ester b).

^1^H and ^13^C{^1^H} NMR spectra for diastereomerically pure di-ester a and b were recorded on a Avance Neo 400 spectrometer (Bruker Biospin AG, Ettlingen, Germany), operating at a frequency of 400.13 MHz. 2-dimensional NMR experiments (COSY, HSQC, and HMBC) were performed for characterizing molecular structures. IR spectra were measured on a Jasco FT/IR-4600 spectrometer (Jasco, Pfungstadt, Germany) equipped with a GladiATR monolithic diamond module (PIKE Technologies, Madison, USA). MS analyses were performed on a Bruker Maxis 4G instrument (Bruker Daltonics, Bremen, Germany) with an ESI system in positive mode.

*Chemical synthesis and purification of CoA thioesters.* 5,6,7,8-tetrahydro-2-naphthoyl-CoA and the CoA thioesters of racemic *cis*- and *trans*-isomer of 2-(carboxymethyl)cyclohexane-1-carboxylic acid were synthesized from the free acids via carbonyldiimidazole activation (Kawaguchi et al., 1981). 26 μmol of carbonyldiimidazole and 32 μmol of the free acid were dissolved in 200 μL anoxic tetrahydrofuran to produce 5,6,7,8-tetrahydro-2-naphthoylimidazole, 2-(carboxymethyl)cyclohexane-1-carboxylimidazole or 2-carboxycyclohexylacetylimidazole. After incubation in 1.5-mL reaction tubes for 2 h at 900 rpm and 22 ℃ in a ThermoMixer C (Eppendorf) in the glove box with an N_2_ atmosphere, 6.5 μmol coenzyme A (CoA-SH) was dissolved in 250 μL 100 mM NaHCO_3_, added to the tetrahydrofuran solution and incubated for 2-4 h. The reaction mixture was titrated to pH 3 with 20% (v/v) formic acid and extracted three times with 500 μL ethylacetate. The aqueous phase was frozen at -70 ℃ and lyophilized in a freeze-dryer (Alpha 2-4 LSC, Martin Christ, Osterode, Germany). CoA thioesters were purified with Chromabond C18 Hydra SPE columns (Macherey-Nagel, Düren, Germany) as described earlier (Weyrauch et al., 2017). Synthesized CoA thioesters were identified and analysed with LC-MS (see main manuscript).

*Synthesis of natural intermediate via biotransformation.* The 2-(carboxymethyl)cyclohexane-1-carboxylic acid CoA thioesters intermediate (m/z = 936) was also synthesized anaerobically from THN-CoA with N47 cell free extract via a THN-CoA reductase assay with NADPH as electron donor (Weyrauch et al., 2020). The assay mixture was amended with 0.1 mM Coenzyme A (CoA-SH) to yield the maximum amount of the intermediate with m/z = 936. The 600 μL reaction mixture contained 100 mM MOPS/KOH buffer, pH 7.3 with 15 mM MgCl_2,_ 0.15 mM THN-CoA, 5 mM ATP, 0.1 mM CoA-SH, 5 mM NADPH, 5 mM NAD^+^, and 50% (v/v) of cell free extract. It was incubated in 1.5-mL reaction tubes at 30 ℃ and shaken at 900 rpm in a ThermoMixer C for 90 min in the glove box with an N_2_ atmosphere. 40 μL samples were taken immediately after adding N47 cell free extract and after 90 min of reaction for analysis. The reaction was stopped by adding a double volume of methanol. Precipitated proteins were afterward pelleted by centrifugation for 45 min at 18,200 × g and 4 ℃ and the supernatant was analysed with LC-MS (see main manuscript). The methanol remaining in the supernatant of the original reaction tubes was evaporated in a rotational vacuum concentrator (RVC 2-25, Martin Christ) and the concentrated fraction was frozen at -70 ℃ and lyophilized in a freeze-dryer.

**Absolute configuration determination of *cis*-2-(carboxymethyl)cyclohexane-1-carboxylic acid by VCD spectroscopy and X-ray crystallography**

Infrared spectroscopy (IR) and vibrational circular dichroism (VCD) spectra of the enantiopure non-CoA derivative of the real substrate of dehydrogenase ThnO were recorded on a Bruker Invenio-R/PMA 50 VCD spectrometer (Bruker, Ettlingen, Germany) at 4 cm^−1^ spectral resolution by accumulating 32 scans for the IR and ~40,000 scans (9 hours accumulation time) for VCD. The sample was dissolved in 0.3 M CDCl_3_ and measured using a BaF_2_ IR cell with 100 μm optical path length. In another experiment, 2 equivalents of 7-azaindole (7AI) were added to suppress dimer formation (Grassin et al., 2022). Baseline correction of the VCD spectra was done by subtraction of the spectra of the solvent recorded under identical conditions. Deriving the absolute configuration from the experimental spectra requires the computation of IR and VCD spectra. Therefore, a conformational sampling was carried out based on a systematic search algorithm on force-field level (MMFF) (Thomas, 1996). The obtained conformers were subjected to further geometry optimizations at B3LYP/6-311++g(2d,p)/IEFPCM(CHCl3) level of theory using Gaussian 09 Rev E.01. Structures of the dimers were generated in the same way. Starting structures with two molecules of 7AI were built manually (Grassin et al., 2022). For the final comparison with the experimental data, the IR and VCD spectra were simulated from the single-conformer spectra using the ΔEZPC‐based Boltzmann weights and by assigning a Lorentzian band shape with half‐width at half‐height of 8 cm^−1^ to the computed dipole and rotational strength.

Crystals suitable for single crystal X-ray analysis were obtained after slow evaporation of a solution of the enantiopure non-CoA derivative of the real substrate (compound 27 in Fig. S1) in chloroform-d1 at room temperature over 6 weeks. The X-ray data of single crystal (pale yellow, 0.195 × 0.146 × 0.132 mm^3^) were measured on a Bruker D8 Venture with Photon II detector (mono-chromated Cu_Kα_ radiation, *λ* = 1.54178 Å, mircofocus source) at 100(2) K. The structure was solved by Direct Methods (SHELXS-2013) (Sheldrick, 1990) and refined anisotropically by full-matrix least-squares on *F*^2^ (SHELXL-2017) (Hübschle et al., 2011; Sheldrick, 2015). Absorption corrections were performed semi-empirically from equivalent reflections on basis of multi-scans (Bruker AXS APEX3). Hydrogen atoms were refined using a riding model. The OH hydrogen atoms were refined freely with their OH bond lengths restrained to be equal to 0.85 Å (DFIX). H1 of both residues are disordered over two positions. An unrestrained refinement without a disorder model leads to hydrogen positions half the way between donor and acceptor of the hydrogen bond. The investigated sample was enantiomerically pure (Sohncke space group) and the anomalous dispersion was sufficiently strong to confirm the expected handedness. The Flack parameter x was determined using Parson's method (Parsons and Flack, 2004; Parsons et al., 2013). For more information on X-ray crystallography, see the Table S3. CCDC-2369957 contains the supplementary crystallographic data for this paper. These data can be obtained free of charge from The Cambridge Crystallographic Data Centre via www.ccdc.cam.ac.uk/data_request/cif.

**Ⅳ. Physical data of compounds involved in derivatization and resolution of enantiomers of cis-2-(carboxymethyl)cyclohexane-1-carboxylic acid**

**(±)-*cis*-2****-(carboxymethyl)cyclohexane-1-carboxylic acid (compound 22)**:

**^1^H-NMR (400 MHz, MeOD, 298 K):** δ = 2.69 – 2.66 (m, 1H, H-2), 2.47 – 2.34 (m, 2H, H-4), 2.33 – 2.26 (m, 1H, H-3), 1.86 – 1.56 (m, 5H, H-9 and H-9’, H-6, H-8), 1.57 – 1.48 (m, 1H, H-6’), 1.47 – 1.36 (m, 2H, H‑7). The COOH-protons are not observed due to exchange with deuterium. For H-8 and H-7 the diastereotopic methylene protons could not be observed as separate signals. The diastereotopic methylene protons of H-6 and H-9 could sometimes be observed separately and are always listed as H-6/H-6’ and H-9/H-9’ for the sake of consistency.

**^13^C{^1^H}-NMR (101 MHz, MeOD, 298 K):** δ = 178.1 (C-1), 176.8 (C-5), 45.4 (C-2), 36.9 (C‑4), 35.5 (C‑3), 29.6 (C-6), 27.5 (C-9), 24.5 (C-7), 24.3 (C-8).

**COSY (400 MHz / 400 MHz, MeOD, 298 K):** δ(^1^H) / δ(^1^H) = 2.69 – 2.66/1.86 – 1.56 (H-2/H-9, H-9’), 2.47 – 2.34/2.33 – 2.26 (H-4/H-3), 2.33 – 2.26/2.47 – 2.34 (H‑3/H‑4), 1.86 – 1.56/2.69 – 2.66, 1.57 – 1.48, 1.47 – 1.36 ( H-9 and H-9’/H-2, H-6/H-6’, H-8 and H-6/H-7), 1.57 – 1.48/1.86 – 1.56 (H-6’/H-6), 1.47 – 1.36/1.86 – 1.56 (H‑7/H-8).

**HSQC (400 MHz / 101 MHz, MeOD, 298 K):** δ(^1^H) / δ(^13^C) = 2.69 – 2.66/45.4 (H-2/C-2), 2.47 – 2.34/36.9 (H-4/C-4), 2.33 – 2.26/35.5 (H-3/C-3), 1.86 – 1.56/29.6, 27.5, 24.3 (H-6/C-6, H-9 and H‑9’/C-9, H-8/C-8), 1.57 – 1.48/29.6 (H-6’/C-6), 1.47 – 1.36/24.5 (H‑7/C-7).

**HMBC (400 MHz / 101 MHz, MeOD, 298 K):** δ(^1^H) / δ(^13^C) = 2.69 – 2.66/178.1, 36.9, 29.6, 27.5, 24.5 (H-3/C-1, C-4, C-6, C-9, C-7), 2.47 – 2.34/176.8, 45.4, 35.5, 29.6 (H-4/C-5, C-2, C-3, C-6), (no crosspeaks were observed for H-2), 1.86 – 1.56/45.4, 35.5, 24.5 (H-6 and H-8/C-2, H-9 and H-9’/C-3, C-7), 1.57 – 1.48/45.4, 36.9, 35.5, 24.3 (H-6’/C-2, C-4, C-3, C-8), 1.47 – 1.36/45.4, 29.6, 27.5, 24.3 (H‑7/C-2, C-6, C-9, C-8).

**IR (ATR-FT):** ν̃ = 2979 (m), 2933 (s), 2861 (m), 1701 (s), 1650 (w), 1557 (w), 1539 (w), 1521 (w), 1507 (w), 1455 (w), 1417 (w), 1276 (m), 1261 (m), 940 (w), 762 (s), 751 (s) cm^‑1^.

**(±)-*trans*-(carboxymethyl)cyclohexane-1-carboxylic acid:**

**^1^H-NMR (400 MHz, MeOD, 298 K):** δ = 2.42 (dd, *J*= 14.7, 3.0 Hz, 1H, H-4), 2.14 – 1.97 (m, 3H, H-2, H-3, H-4‘), 1.97 – 1.92 (m, 1H, H-9), 1.92 – 1.85 (m, 1H, H-6), 1.80 – 1.70 (m, 2H, H‑7), 1.55 – 1.42 (m, 1H, H-9’), 1.38 – 1.22 (m, 2H, H-8), 1.13 – 1.00 (m, 1H, H-6’). The COOH-protons are not observed due to exchange with deuterium.

**^13^C{^1^H}-NMR (101 MHz, MeOD, 298 K):** δ = 179.3 (C-1), 176.2 (C-5), 50.2 (C-3), 40.3 (C‑4), 37.2 (C‑2), 32.2 (C-6), 31.3 (C-9), 26.6 (C-8), 26.4 (C-7).

**COSY (400 MHz/ 400 MHz, MeOD, 298 K):** δ(^1^H) / δ(^1^H) = 2.42/2.14 – 1.97 (H-4/H-3, H‑4’), 2.14 – 1.97/2.42, 1.55 – 1.42, 1.13 – 1.10 (H-4’/H-3, H-4 and H-2/H-9’ and H-3/H‑6’), 1.97 – 1.92/1.80 – 1.70, 1.55 – 1.42 (H-9/H-7, H-9’), 1.92 – 1.85/1.80 – 1.70, 1.13 – 1.10 (H‑6/H‑7, H-6’), 1.80 – 1.70/1.97 – 1.92, 1.92 – 1.85, 1.38 – 1.22 (H-7/H-9, H-6, H‑8), 1.55 – 1.42/2.14 – 1.97, 1.97 – 1.92, 1.38 – 1.22 (H-9’/H-2, H-9, H-8), 1.38 – 1.22/1.80 – 1.70, 1.55 – 1.42, 1.13 – 1.00 (H-8/H-7, H-9’, H-6’), 1.13 – 1.00/2.14 – 1.97, 1.92 – 1.85, 1.38 – 1.22 (H-6’/H-2, H-6, H-8).

**HSQC (400 MHz/ 101 MHz, MeOD, 298 K):** δ(^1^H) / δ(^13^C) = 2.42/40.3 (H-4/C-4), 2.14 – 1.97/50.2, 40.3, 37.2 (H-3/C-3 and H-4‘/C-4 and H-2/C-2), 1.97 – 1.92/31.3 (H‑9/C‑9), 1.92 – 1.85/32.2 (H-6/C-6), 1.80 – 1.70/26.4 (H-7/C-7), 1.55 – 1.42/31.3 (H‑9’/C-9), 1.38 – 1.22/26.6 (H-8/C-8), 1.13 – 1.00/32.2 (H-6’/C-6).

**HMBC (400 MHz/ 101 MHz, MeOD, 298 K):** δ(^1^H) / δ(^13^C) = 2.42/176.2, 50.2, 37.2, 32.2 (H-4/C‑5, C‑3, C-2, C-6), 2.14 – 1.97/179.3, 176.2, 50.2, 40.3, 37.2, 32.2 (H-3/C-1, C‑5, C-6 and H-2/C-4 and H‑4’/C‑5, C-2), 1.97 – 1.92/50.2, 26.4 (H-9/C3, C-7), 1.92 – 1.85/40.3, 37.2 (H-6/C-4, C‑2), 1.80 – 1.70/50.2, 37.2, 32.2 (H-7/C-3, C-2, C-6), 1.55 – 1.42/179.3, 50.2, 37.2, 26.4 (H-9’/C‑1, C‑3, C-2, C-7), 1.38 – 1.22/37.2, 32.2, 26.6 (H-8/C‑2, C-6, C-8), 1.13 – 1.00/40.3, 37.2, 26.6 (H‑6’/C-4, C-2, C-8).

**IR (ATR-FT):** ν̃ = 2927 (m), 2848 (m), 2648 (w), 2574 (w), 1713 (s), 1689 (s), 1448 (w), 1427 (m), 1406 (m), 1343 (w), 1291 (m), 1239 (s), 1188 (m), 1114 (w), 1087 (w), 1047 (w), 920 (m), 893 (m), 848 (w), 826 (w), 679 (m), 531 (w) cm^-1^.

**(*R*,*R*,*R*,*R*)-2-phenylpropyl-2-(2-oxo-2-(-2-phenylpropoxy)ethyl)cyclohexane-1-carboxylate (compound 25):**

**^1^H-NMR (400 MHz, MeOD, 298 K):** δ = 7.31 – 7.10 (m, 10H, H-Aryl), 4.25 – 4.03 (m, 4H, H-E1 and H‑E1‘), 3.15 – 3.00 (m, 2H H-E2 and H-E2‘), 2.46 (dt, *J* = 6.8, 4.2 Hz, 1H, H-2), 2.27 – 2.03 (m, 3H, H-3 and H-4), 1.66 – 1.56 (m, 1H, H-9), 1.52 – 1.20 (13H, m, H-6, H-6‘, H-9‘, H-7, H-8, H-E3, H‑E3‘).

**^13^C{^1^H}-NMR (101 MHz, MeOD, 298 K):** δ = 175.7 (C-1), 174.5 (C-5), 144.9 (2 signals, 2x C-Aryl_quart_), 129.7 (C-Aryl), 128.5 (2 signals, 2x C-Aryl), 127.8 (C-Aryl), 70.4 and 70.2 (C‑E1 and C-E1’), 45.5 (C-2), 40.5 (2 signals, C-E2 and C-E2‘), 37.5 (C-4), 35.8 (C-3), 29.6 (C-6), 27.8 (C-9), 24.6 and 24.3 (C-7 and C‑8), 18.8 and 18.7 (C-E3 and C-E3’).

**COSY (400 MHz / 400 MHz, MeOD, 298 K):** δ(^1^H) / δ(^1^H) = 4.25 – 4.03/3.15 – 3.00 (H‑E1 and H-E1‘/H-E2 and H-E2‘), 3.15 – 3.00 /4.25 – 4.03, 1.52 – 1.20 (H-E2 and H-E2‘/H-E1 and H-E1‘, H-E3 and H-E3‘), 2.46/1.66 – 1.56, 1.52 – 1.20 (H-2/H-9, H-9‘, H-8), 2.27 – 2.03/1.52 – 1.20 (H-3/H-6), 1.66 – 1.56/2.46, 1.52 – 1.20 (H-9/H-2, H-9‘, H-8), 1.52 – 1.20/3.15 – 3.00, 2.46, 2.27 – 2.03, 1.66 – 1.56 (H-E3 and H‑E3‘/H-E2 and H‑E2‘, H-6‘, H‑8/H-2, H‑3, H-9).

**HSQC (400 MHz / 101 MHz, MeOD, 298 K):** δ(^1^H) / δ(^13^C) = 7.31 – 7.10/129.7, 128.5, 127.8 (H‑Aryl/C-Aryl), 4.25 – 4.03/70.4 and 70.2 (H-E1/C-E1 and H‑E1’/C‑E1’), 3.15 – 3.00/40.5 (H-E2/C-E2 and H‑E2’/C‑E2’), 2.46/45.5 (H-2/C-2), 2.27 – 2.03/37.5, 35.8 (H‑4/C-4 and H-3/C-3), 1.66 – 1.56/27.8 (H‑9/C‑9), 1.52 – 1.20/29.6, 27.8, 24.6, 24.3, 18.8 and 18.7 (H-6/C-6, H‑9‘/C-9‘, H-7 and H-8/C-7 and C-8, H-E3 and H-E3‘/C-E3 and C‑E3‘).

**HMBC (400 MHz / 101 MHz, MeOD, 298 K):** δ(^1^H) / δ(^13^C) = 7.31 – 7.10/144.9, 129.7, 128.5, 127.8 (H-Aryl/C-Aryl_quart_, H-Aryl/C-Aryl), 4.25 – 4.03/175.7, 174.5, 144.9, 40.5, 18.8 and 18.7 (H-E1 and H-E1’/C-1, C-5, C-Aryl_quart_, C-E2 and C-E2‘, C-E3 and C-E3‘), 3.15 – 3.00/144.9, 128.5, 70.4 and 70.2, 18.8 and 18.7 (H-E2 and H-E2‘/C-Aryl_quart_, C-Aryl, C-E1 and C-E2‘, C-E3 and C-E3‘), 2.46/175.7, 37.5, 35.8, 29.6, 27.8, 24.6 and 24.3 (H-2/C-1, C‑4, C-3, C-6, C‑9, C-7 and C-8), 2.27 – 2.03/174.5, 45.5, 35.8, 29.6 (H-3/C-5 and H-4/C-2, C-3, C-6), 1.52 – 1.20/144.9, 70.4 and 70.2, 40.5 (H-E3 and H-E3‘/C‑Aryl_quart_ , C-E1 and C‑E1‘, C-E2 and C-E2‘).

**IR (ATR-FT):** ν̃ = 3059 (w), 3029 (w), 2968 (s), 2932 (s), 2861 (m), 1729 (s), 1494 (w), 1453 (m), 1389 (w), 1376 (w), 1287 (w), 1246 (w), 1162 (s), 1126 (w), 1005 (w), 762 (w) 700 (m) cm^-1^.

**MS (ESI-pos, MeOH):** m/z = 445.2354 ([M+Na]^+^, calcd. 445.2349 for [C_27_H_34_O_4_Na]^+^); 423.2532 ([M+H]^+^, calcd. 423.2530 for [C_27_H_35_O_4_]^+^).

**(*S*,*S*,*R*,*R*)-2-phenylpropyl-2-(2-oxo-2-(-2-phenylpropoxy)ethyl)cyclohexane-1-carboxylate (compound 26):**

**^1^H-NMR (400 MHz, MeOD, 298 K):** δ = 7.36 – 7.09 (m, 10H, H-Aryl), 4.32 – 4.04 (m, 4H, H-E1 and H‑E1‘), 3.12 – 3.00 (hd, *J* = 7.1, 2.4 Hz, 2H, H-E2 and H-E2‘), 2.51 (dt, *J* = 7.9, 4.0 Hz, 1H, H-2), 2.28 – 1.99 (m, 3H, H-3 and H-4), 1.70 – 1.60 (m, 1H, H-­9), 1.56 – 1.40 (m, 4H, H-9‘, H-6, H-7), 1.38 – 1.22 (m, 9H, H‑6‘, H-8, H-E3, H-E3‘).

^13^C{^1^H}-NMR (101 MHz, MeOD, 298 K): δ = 175.6 (C-1), 174.3 (C-5), 144.7 (C-Aryl_quart_), 129.5 (C‑Aryl), 128.4 (C-Aryl), 127.7 (C-Aryl), 70.3 and 70.0 (C-E1 and C-E1‘), 45.4 (C-2), 40.3 (2 signals, C‑E2 and C-E2‘), 37.3, (C-4), 35.6 (C-3), 29.5 (C-6), 27.5 (C-9), 24.4 and 24.2 (C-8 and C-7), 18.7 and 18.6 (C­E3 and C-E3’).

**COSY (400 MHz / 400 MHz, MeOD, 298 K):** δ(^1^H) / δ(^1^H) = 4.32 – 4.04/3.12 – 3.00 (H‑E1/H-E2 and H‑E1‘/H‑E2‘), 3.12 – 3.00/4.32 – 4.04, 1.38 – 1.22 (H-E2/H-E1, H-E3 and H‑E2‘/H-E1‘, H-E3‘), 2.51/2.28 – 1.99, 1.70 – 1.60, 1.56 – 1.40 (H-2/H-3, H-9, H-9‘), 2.28 – 1.99/2.51, 1.56 – 1.40, 1.38 – 1.22 (H-3/H-2, H-6, H-6‘), 1.70 – 1.60/2.51, 1.56 – 1.40, 1.38 – 1.22 (H-9/H-2, H-9‘, H-8), 1.56 – 1.40/2.51, 2.28 – 1.99, 1.70 – 1.60, 1.38 – 1.22 (H‑9‘/H-2 and H-6/H-3 and H-9’/H-9, H-8), 1.38 – 1.22/3.12 – 3.00, 2.28 – 1.99, 1.70 – 1.60, 1.38 – 1.22 (H-3/H-E2 and H-E3‘/H-E2‘ and H-6‘/H-3 and H‑8/H-9, H-7).

**HSQC (400 MHz / 101 MHz, MeOD, 298 K):** δ(^1^H) / δ(^13^C) = 7.36 – 7.09/129.5, 128.4, 127.7 (H‑Aryl/C‑Aryl), 4.32 – 4.04/70.3, 70.0 (H-E1/C-E1 and H-E1‘/C-E1‘), 3.12 – 3.00/40.3 (H-E2/C-E2 and H‑E2‘/C-E2‘), 2.51/45.4 (H-2/C-2), 2.28 – 1.99/37.3, 35.6 (H‑4/C-4 and H-3/C-3), 1.70 – 1.60/27.5 (H‑9/C-9), 1.56 – 1.40/29.5, 27.5, 24.4 and 24.2 (H-6/C-6 and H-9’/C-9, H-7/C-7), 1.38 – 1.22/29.5, 24.4 and 24.2, 18.7 and 18.6 (H‑6‘/C‑6, H-8/C-8 and H-E3, H-E3‘/C-E3, C-E3‘).

**HMBC (400 MHz / 101 MHz, MeOD, 298 K):** δ(^1^H) / δ(^13^C) = 7.36 – 7.09/144.7, 129.5, 128.4, 127.7 (H‑Aryl/C-Aryl_quart_, C-Aryl), 4.32 – 4.04/175.6, 174.3, 144.7, 40.3, 18.7 and 18.6 (H-E1 and H-E1‘/C-1, C‑5, C-Aryl_quart_, C-E2, C-E2‘, C-E3, C-E3‘), 3.12 – 3.00/144.7, 128.4, 70.3, 70.0, 18.7 and 18.6 (H-E2 and H‑E2‘/CAryl_quart_, C-Aryl, C-E1, C-E1‘, C-E3, C‑E3‘), 2.51/175.6, 37.3, 35.6, 29.5, 27.5, 24.4 and 24.2 (H-2/ C-1, C-4, C-3, C-6, C-9, C-7 and C-8), 2.28 – 1.99/174.3, 45.4, 35.6, 29.5 (H-3/C-5, H-4/C-2, C-3, C-6), 1.70 – 1.60/24.4 and 24.2 (H-9/C-7), 1.56 – 1.40/45.4, 24.4 and 24.2 (H-6/C-2, C-8 and H-9’/C-7), 1.38 – 1.22/144.7, 70.3 and 70.0, 45.4, 40.3 (H-E3 and H-E3‘/C-Aryl_quart_, C-E1 and C-E1‘, H-6‘/C-2, H-E3 and H-E3‘/C-E2 and C-E2‘).

**IR (ATR-FT):** ν̃ = 3063 (w), 3028 (m), 2967 (s), 2931 (s), 2859 (m), 1727 (s), 1605 (w), 1494 (w), 1452 (m), 1390 (w), 1375 (w), 1277 (m), 1260 (m), 1210 (w), 1158 (s), 1124 (m), 1023 (m), 1002 (m), 761 (s), 699 (s), 534 (w) cm^-1^.

**MS (ESI-pos, MeOH):** m/z = 445.2356 ([M+Na]^+^, calcd. 445.2349 for [C_27_H_34_O_4_Na]^+^); 423.2533 ([M+H]^+^, calcd. 423.2530 for [C_27_H_35_O_4_]^+^).

**Ⅴ. ^1^H NMR and ^13^C{^1^H} NMR spectra of compounds involved in derivatization and resolution of enantiomers of cis-2-(carboxymethyl)cyclohexane-1-carboxylic acid**

1. ^1^H-NMR spectrum of *cis*-*rac*-2-(carboxymethyl)cyclohexane-1-carboxylic acid (compound 22) (400 MHz, 298 K, MeOD).

2. ^13^C{^1^H}-NMR spectrum of *cis*-*rac*-2-(carboxymethyl)cyclohexane-1-carboxylic acid (compound 22) (101 MHz, 298 K, MeOD).

3. ^1^H-NMR spectrum of *trans*-*rac*-2-(carboxymethyl)cyclohexane-1-carboxylic acid (400 MHz, 298 K, MeOD).

4. ^13^C{^1^H}-NMR spectrum of *trans*-*rac*-2-(carboxymethyl)cyclohexane-1-carboxylic acid (101 MHz, 298 K, MeOD).

5. ^1^H-NMR spectrum of (*R*,*R*,*R*,*R*)-2-phenylpropyl-2-(2-oxo-2-(-2-phenylpropoxy)ethyl)cyclohexane-1-carboxylate (compound 25) (400 MHz, 298 K, MeOD).

6. ^13^C{^1^H}-NMR spectrum of (*R*,*R*,*R*,*R*)-2-phenylpropyl-2-(2-oxo-2-(-2-phenylpropoxy)ethyl)cyclohexane-1-carboxylate (compound 25) (101 MHz, 298 K, MeOD).

7. ^1^H-NMR spectrum of (*S*,*S*,*R*,*R*)-2-phenylpropyl-2-(2-oxo-2-(-2-phenylpropoxy)ethyl)cyclohexane-1-carboxylate (compound 26) (400 MHz, 298 K, MeOD).

8. ^13^C{^1^H}-NMR spectrum of (*S*,*S*,*R*,*R*)- 2-phenylpropyl-2-(2-oxo-2-(-2-phenylpropoxy)ethyl)cyclohexane-1-carboxylate (compound 26) (101 MHz, 298 K, MeOD).

9. ^1^H-NMR spectrum of (1*R*,2*R*)-2-(carboxymethyl)cyclohexane-1-carboxylic acid (compound 27) (400 MHz, 298 K, MeOD).

10. ^1^H-NMR spectrum of (1*S*,2*S*)-2-(carboxymethyl)cyclohexane-1-carboxylic acid (compound 28) (400 MHz, 298 K, MeOD).
